# Mutant P53 induces MELK expression by release of wild-type P53-dependent suppression of FOXM1

**DOI:** 10.1038/s41523-019-0143-5

**Published:** 2020-01-03

**Authors:** Lakshmi Reddy Bollu, Jonathan Shepherd, Dekuang Zhao, Yanxia Ma, William Tahaney, Corey Speers, Abhijit Mazumdar, Gordon B. Mills, Powel H. Brown

**Affiliations:** 1 Department of Clinical Cancer Prevention, The University of Texas MD Anderson Cancer Center, Houston, Texas USA; 2 Department of Molecular and Cellular Biology, Baylor College of Medicine, Houston, Texas USA; 3 Department of Radiation Oncology, University of Michigan, Ann Arbor, Michigan USA; 4 Department of Systems Biology, The University of Texas MD Anderson Cancer Center, Houston, Texas USA; 5 Present Address: Precision Oncology, OHSU Knight Cancer Institute, Oregon Health and Science University, 2720 Southwest Moody Avenue, Knight Cancer Research Building, Level 2, Portland, Oregon 97201 USA

**Keywords:** Breast cancer, Transcription

## Abstract

Triple-negative breast cancer (TNBC) is the most aggressive form of breast cancer, and is associated with a poor prognosis due to frequent distant metastasis and lack of effective targeted therapies. Previously, we identified maternal embryonic leucine zipper kinase (MELK) to be highly expressed in TNBCs as compared with ER-positive breast cancers. Here we determined the molecular mechanism by which MELK is overexpressed in TNBCs. Analysis of publicly available data sets revealed that MELK mRNA is elevated in p53-mutant breast cancers. Consistent with this observation, MELK protein levels are higher in p53-mutant vs. p53 wild-type breast cancer cells. Furthermore, inactivation of wild-type p53, by loss or mutation of the p53 gene, increases MELK expression, whereas overexpression of wild-type p53 in p53-null cells reduces MELK promoter activity and MELK expression. We further analyzed MELK expression in breast cancer data sets and compared that with known wild-type p53 target genes. This analysis revealed that MELK expression strongly correlates with genes known to be suppressed by wild-type p53. Promoter deletion studies identified a p53-responsive region within the MELK promoter that did not map to the p53 consensus response elements, but to a region containing a FOXM1-binding site. Consistent with this result, knockdown of FOXM1 reduced MELK expression in p53-mutant TNBC cells and expression of wild-type p53 reduced FOXM1 expression. ChIP assays demonstrated that expression of wild-type p53 reduces binding of E2F1 (a critical transcription factor controlling FOXM1 expression) to the FOXM1 promoter, thereby, reducing FOXM1 expression. These results show that wild-type p53 suppresses FOXM1 expression, and thus MELK expression, through indirect mechanisms. Overall, these studies demonstrate that wild-type p53 represses MELK expression by inhibiting E2F1A-dependent transcription of FOXM1 and that mutation-driven loss of wild-type p53, which frequently occurs in TNBCs, induces MELK expression by suppressing FOXM1 expression and activity in p53-mutant breast cancers.

## Introduction

Triple-negative breast cancers (TNBCs), a breast cancer subtype, are highly aggressive tumors occurring frequently in young women and in African American women who have a very poor prognosis. TNBCs are defined as breast cancers lacking the expression of estrogen receptor (ER), progesterone receptor, and epidermal growth factor receptor 2 (HER2). In our previous studies, we identified several kinases overexpressed in TNBCs as compared with ER-positive breast cancers and showed that inhibition of the expression of several of these kinases suppressed the growth of TNBC cells.^1^ One such critical kinase is the maternal embryonic leucine zipper kinase (MELK).^1^ Several reports have identified that MELK expression is highly elevated in many human cancers and high MELK expression is associated with poor prognosis.^2–7^

MELK is a serine/threonine kinase belonging to the AMPK family of kinases known to regulate cellular metabolism.^8^ MELK was initially discovered as one of the three mRNAs uniquely expressed during early embryonic development.^9,10^ MELK is also one of the proliferation markers included in Food and Drug Administration-approved breast cancer prognostic panels, such as MammaPrint^11,12^ and PAM50,^13^ which are used to plan treatment for breast cancer patients and to predict cancer recurrence. High expression of MELK is required for maintenance of mammary tumor-initiating cells^14^ and glioma stem cells.^15,16^ In addition, MELK has been reported to control several biological processes including cell proliferation and cell cycle, invasion, apoptosis, splicing, and resistance to chemotherapy and radiation therapy.^17–28^ In contrast, MELK expression is not required for normal development, as knockout mice have no developmental defects.^24^ However, two recent studies showed that knockout of MELK, using CRISPR-cas9 system, did not reduce the growth of TNBC cells.^29,30^ Further studies by Wang et al.^31,32^ addressed the discrepancy of MELK role on cancer cell growth, which revealed that MELK expression is required for clonogenic growth of TNBC cells when cells were cultured at low density, whereas MELK is not required for clonogenic growth of ER-positive breast cancer cells that have low MELK expression. In this study, we sought to determine the molecular mechanism by which MELK is upregulated in TNBC breast cancers. We discovered that MELK expression is associated with p53 mutation status. The tumor suppressor protein, p53, is a transcription factor that controls both activation and repression of gene expression in eukaryotic cells.^33,34^ Wild-type p53 (WT p53) regulates the expression of many genes by inducing or repressing transcription, whereas loss or mutation of p53 promotes tumorigenesis.^33–35^ Inactivation of WT p53 is a common event during cancer development and nearly half of all human cancers, and over 80% of TNBC patients, harbor mutations in the p53 gene, resulting in loss of WT p53 activity.^36^

Through these studies, we elucidated the molecular mechanism by which MELK is highly expressed in TNBCs. We discovered that high expression of MELK in p53-mutant breast cancers is due to the loss of WT p53 activity, which normally functions in p53 WT cells by repressing E2F1A binding to the FOXM1 promoter, which reduces FOXM1 expression and, in turn, MELK expression. We also discovered that in p53-mutant TNBCs, E2F1A is recruited to FOXM1 promoter, causing increased expression of FOXM1, which binds to the MELK promoter and activates MELK transcription, thus increasing MELK expression in TNBCs. These studies demonstrate that in p53-mutant breast cancers, E2F1A and FOXM1 regulates the expression of MELK, a novel kinase associated with poor prognosis and a potential novel target for the treatment of these aggressive cancers.

## Results

### MELK expression is highly elevated in p53-mutant breast cancers

**Fig. 1:**
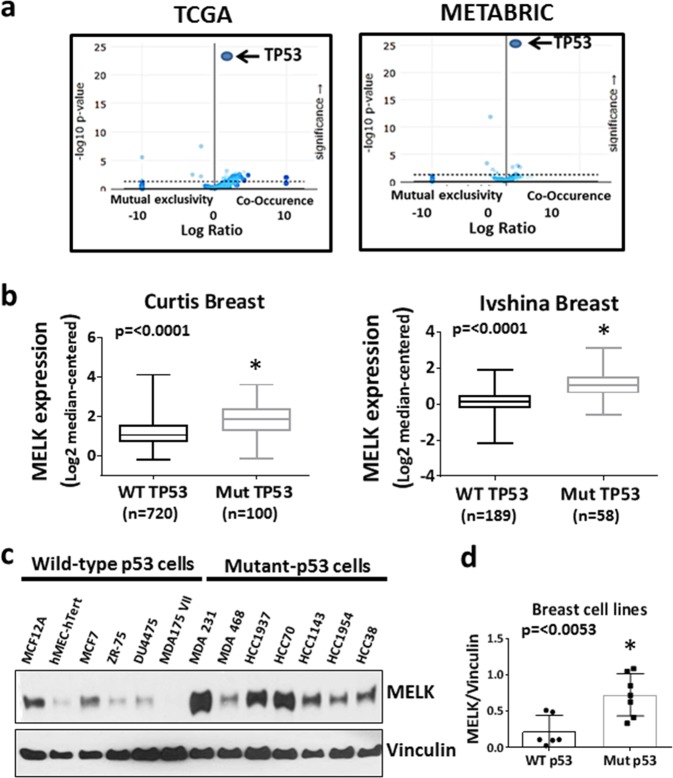
Correlation between MELK expression and p53 mutation status. **a** Analysis of breast cancer data sets (TCGA and METABRIC) to determine the co-occurrence of high MELK expression with oncogenic mutations in breast cancer patients. High MELK expression was defined as a microarray *z*-score of > 2.0. **b** Comparison of MELK mRNA expression between p53 wild-type and p53-mutant breast cancer patient samples in breast cancer data sets. The scale for MELK expression is log2-median-centered ratio. Error bars represents a range of minimum to maximum log2-median-centered MELK expression levels. The box encompasses the upper and lower quartiles. The central line represents the median. **c** Western blotting analysis of MELK protein in a panel of breast cell lines with known p53 mutation status. Vinculin was used as a loading control. **d** Comparison of normalized MELK protein levels between wild-type and mutant p53 breast cell lines. *Statistical significance of *p*-value < 0.05. Error bars represents ± SE in **d**.

We previously identified protein kinases upregulated in human ER-negative tumors as compared with ER-positive breast cancers. One of these kinases, MELK, is highly expressed in TNBC tumors compared with normal breast and non-TNBC breast cancers (Supplementary Fig. 1A, B), and the elevation of MELK expression occurs in early-stage breast cancers (Supplementary Fig. 1C, D). To investigate the mechanism by which MELK expression is highly elevated in TNBCs, we analyzed The Cancer Genome Atlas (TCGA) and Molecular Taxonomy of Breast Cancer International Consortium (METABRIC) breast cancer patient data sets (cBioPortal) to determine whether upregulated MELK expression correlates with gene copy number, promoter methylation, or any specific oncogenic mutations. Our analysis revealed that elevated MELK expression is significantly enriched in breast tumors with p53 mutations in both TCGA Provisional and METABRIC^37^ data sets (Fig. 1a). Consistent with our finding, upregulation of MELK expression in p53-mutant breast tumors compared with their matched normal breast tissues is significantly higher than WT p53 counterparts (Supplementary Fig. 2A). Nearly 80% of TNBC tumors harbor mutations in the p53 gene, a tumor suppressor gene, which is inactivated in over 50% of all human cancers. Furthermore, analysis of two independent breast cancer patient data sets (Curtis and Ivshina)^38,39^ showed that MELK expression is elevated at mRNA levels in p53-mutant breast cancer tumors (Fig. 1b) and the elevation of MELK expression is independent of ER status (Supplementary Fig. 2B). Consistent with this observation, MELK expression is significantly elevated in p53-mutant cancers of the lung, bladder, brain, and prostate compared with their WT p53 tumors (Supplementary Fig. 2C). Similar to MELK mRNA expression, MELK protein is elevated in p53-mutant breast cancer cell lines compared with WT p53 cells (Fig. 1c, d). Minn et al.^40^ showed that MELK is one of the 54 genes associated with metastasis of primary breast cancer cells to the lungs. To determine whether MELK expression status correlates with metastasis-free survival rate, we analyzed three independent breast cancer data sets (Schmidt, Desmedt, and Esserman),^41–43^ which demonstrated that high MELK expression is significantly associated with poor metastasis-free survival in all three data sets (Supplementary Fig. 1E).

### Dominant-negative p53 mutants increase MELK expression by inhibiting WT p53

**Fig. 2:**
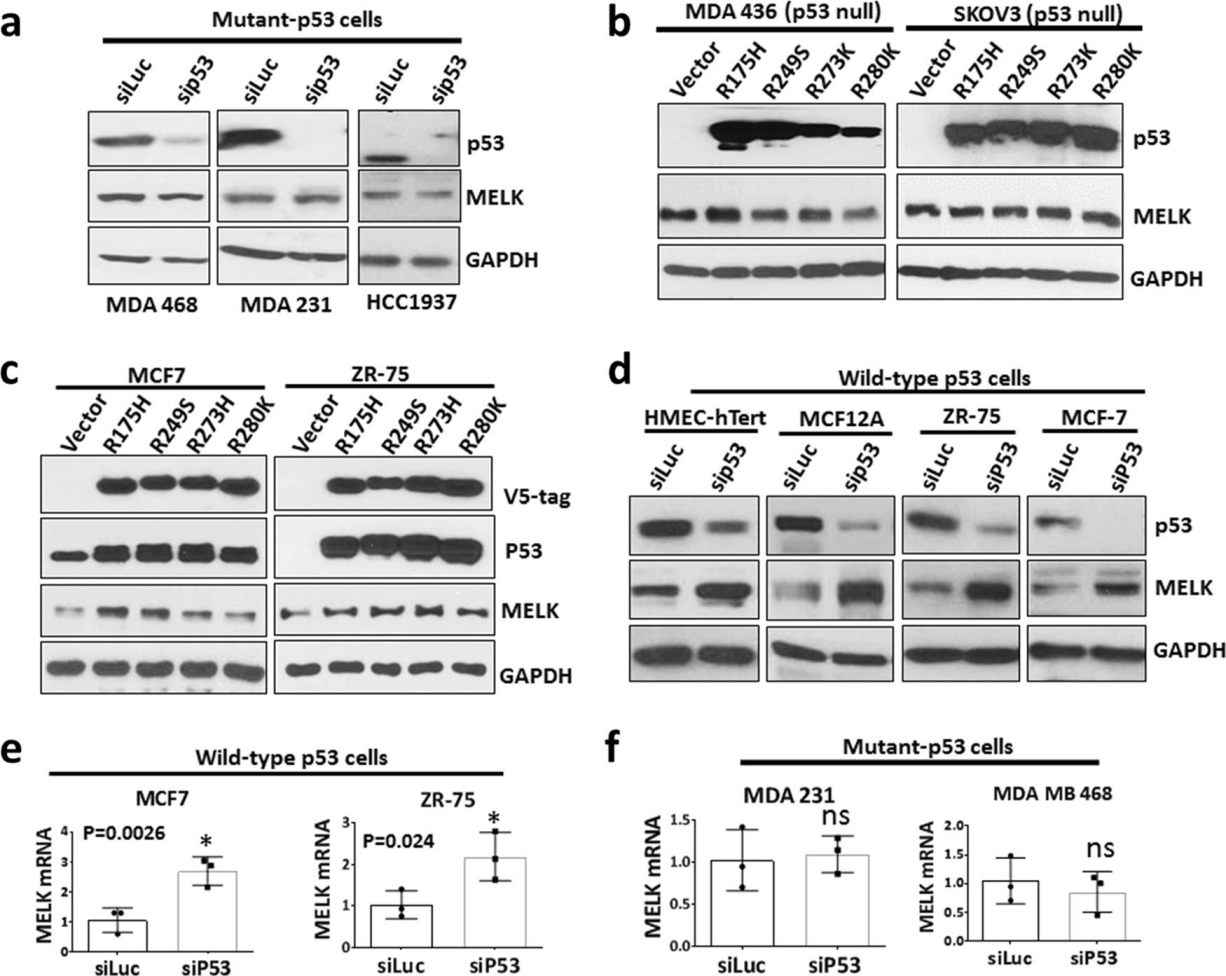
Loss of wild-type p53 increases MELK expression in breast cancer cells. **a** Western blotting analysis of MELK and p53 protein levels in TNBC cells (MDA 468, MDA 231, and HCC1937) after 48 h of p53 knockdown using siRNA. HCC1937 cells have a truncation mutation in p53 resulting in low molecular weight band for p53. **b** Western blotting analysis of MELK protein levels in p53-null cells (MDA 436 (breast) and SKOV3 (ovarian)) stably expressing dominant-negative p53 mutants. **c** Western blotting analysis of MELK protein levels in MCF7 and ZR-75 (express wild-type p53) cells stably expressing dominant-negative p53 mutants. V5-tag detects ectopically expressed p53 mutants. **d** Western blotting analysis of MELK and p53 protein levels in wild-type p53-expressing cells (HMEC-hTert, MCF12A, ZR-75, and MCF7) after 48 h of p53 knockdown using siRNA. GAPDH was used as a loading control. **e** qRT-PCR analysis of MELK mRNA levels in wild-type p53-expressing cells (MCF7 and ZR-75) after 48 h of p53 knockdown using siRNA. **f** qRT-PCR analysis of MELK mRNA levels in p53-mutant-expressing cells (MDA 231 and MDA 468) after 48 h of p53 knockdown using siRNA. *Statistical significance of *p*-value < 0.05. Error bars represent ± SD.

Previous reports have shown that gain-of-function p53 mutants induce the expression of several critical genes involved cancer initiation and progression.^35,44^ We next tested whether p53 mutants regulate MELK expression by depleting the expression of mutant p53 in TNBC cells that have high MELK expression. Depletion of mutant p53 using small interfering RNA (siRNA) did not reduce the expression of MELK (Fig. 2a). To determine whether mutant p53 induces the expression of MELK, we generated stable cells to express p53 mutants (R175H, R249S, R273H, and R280K) in p53-null cells (MDA MB 436 and SKOV3) and measured MELK expression. In both SKOV3 and MDA MB 436 cells, overexpression of p53 mutants did not induce MELK expression (Fig. 2b). However, overexpression of these p53 mutants in WT p53 cells (MCF7 and ZR-75 cells) induced MELK expression (Fig. 2c). These mutants appear to act as dominant-negative proteins, which inhibit the WT activity of p53. These data suggest that inhibition of WT p53 is the key mechanism to elevate MELK expression.

### Inactivation of WT p53 increases MELK expression

**Fig. 3:**
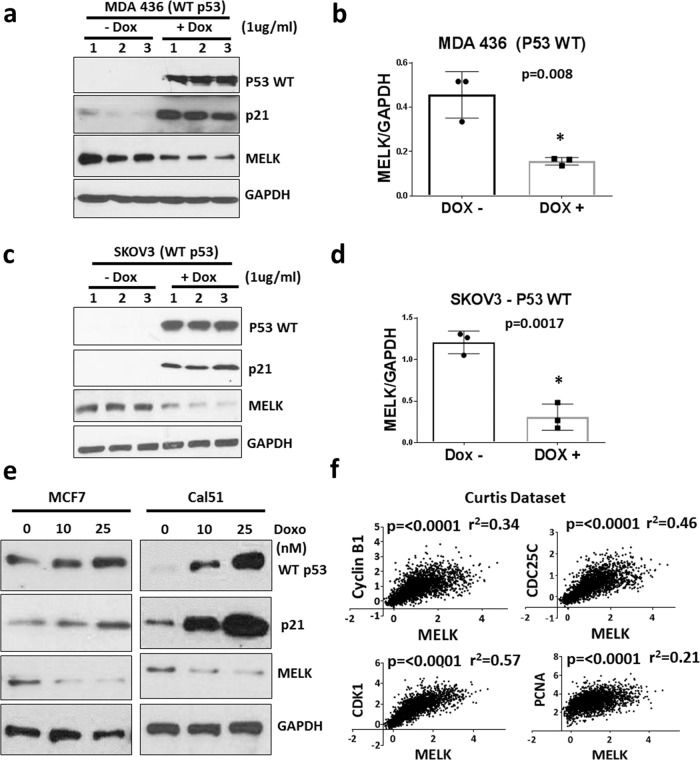
Wild-type p53 represses MELK expression. Western blotting analysis of MELK and p53 protein levels in p53-null cells (MDA MB 436 (**a**) and SKOV3 (**c**)) after inducing wild-type p53 for 48 h. Wild-type p53 was induced using a doxycycline-inducible system, in p53-null cells. p21 expression was used to demonstrate the functional activity of wild-type p53 and GAPDH was used as a loading control. Numbers 1, 2, and 3 indicates triplicates of protein samples in **a**, **c**. MELK protein levels from **a** and **c** were normalized to GAPDH levels and were plotted for MDA MB 436 (**b**) and SKOV3 (**d**) cells. **e** Western blotting analysis of MELK, p21, and p53 protein levels in MCF7 and Cal51 cells after inducing wild-type p53 with doxorubicin (“Doxo”) at increasing concentrations (0, 10, and 25 nM) for 48 h. **f** Analysis of breast cancer dataset to determine the correlation between MELK mRNA levels and wild-type p53-repressed genes in the Curtis breast cancer dataset. The scale for the expression of MELK (*X*-axis) and p53-repressed genes (*Y*-axis) is log2-median-centered ratio. The Pearson’s correlation *p*-values and *r*^2^-values were calculated using GraphPad prism. *Statistical significance of *p*-value < 0.05. Error bars represent ± SD.

We next tested whether elevated expression of MELK is due to the loss of WT activity of p53. To test this hypothesis, we inhibited WT p53 by using siRNA in WT p53-containing cells. As shown in Fig. 2d, we depleted WT p53 in two normal breast epithelial cell lines and two ER-positive breast cancer cell lines that have WT p53. Knockdown of WT p53 significantly increased MELK protein expression. Similarly, knockdown of p53 increased MELK mRNA expression in WT p53 cells (Fig. 2e) but not in mutant p53 cells (Fig. 2f). These results suggest that loss of WT activity of p53 is the key mechanism for elevated MELK expression in TNBC cells. Based on this data, we hypothesized that WT p53 acts as a transcriptional repressor of MELK. To test this hypothesis, we generated stable MDA MB 436 and SKOV3 cells with inducible expression of WT p53 using a doxycycline (Dox)-inducible system (pInducer20-p53 WT). SKOV3 and MDA MB 436 are p53-null cell lines and express high levels of MELK. As shown in Fig. 3a–d, induction of WT p53 by Dox treatment significantly reduced MELK protein levels in both cell lines, while increasing p21 protein levels (a downstream marker of WT p53). In addition, Dox treatment did not reduce MELK protein levels in MDA MB 436 cells expressing empty vector, suggesting that Dox reduces MELK expression by inducing WT p53 (Supplementary Fig. 3A). Furthermore, inhibition of the proteasome-dependent protein degradation pathway did not affect the repressive effects of WT p53 on MELK protein levels (Supplementary Fig. 3B). In addition, induction of WT p53 by doxorubicin in MCF7 and Cal51 (WT p53 cells) repressed MELK protein levels in a dose-dependent manner Fig. 3e. In addition to transcriptional activation, WT p53 represses gene transcription. We analyzed breast cancer data sets (Curtis and TCGA) to determine whether MELK expression correlates with WT p53-induced or repressed genes.^45^ Analysis of breast cancer data sets revealed that MELK expression is significantly correlated with p53-repressed genes (Fig. 3f and Supplementary Fig. 3C) but not with p53-induced genes (Supplementary Fig. 3D). Collectively, these results suggest that WT p53 acts as a negative regulator of MELK expression, and that loss of WT p53 activity elevates MELK expression in TNBC cells.

### WT p53 represses MELK promoter activity

**Fig. 4:**
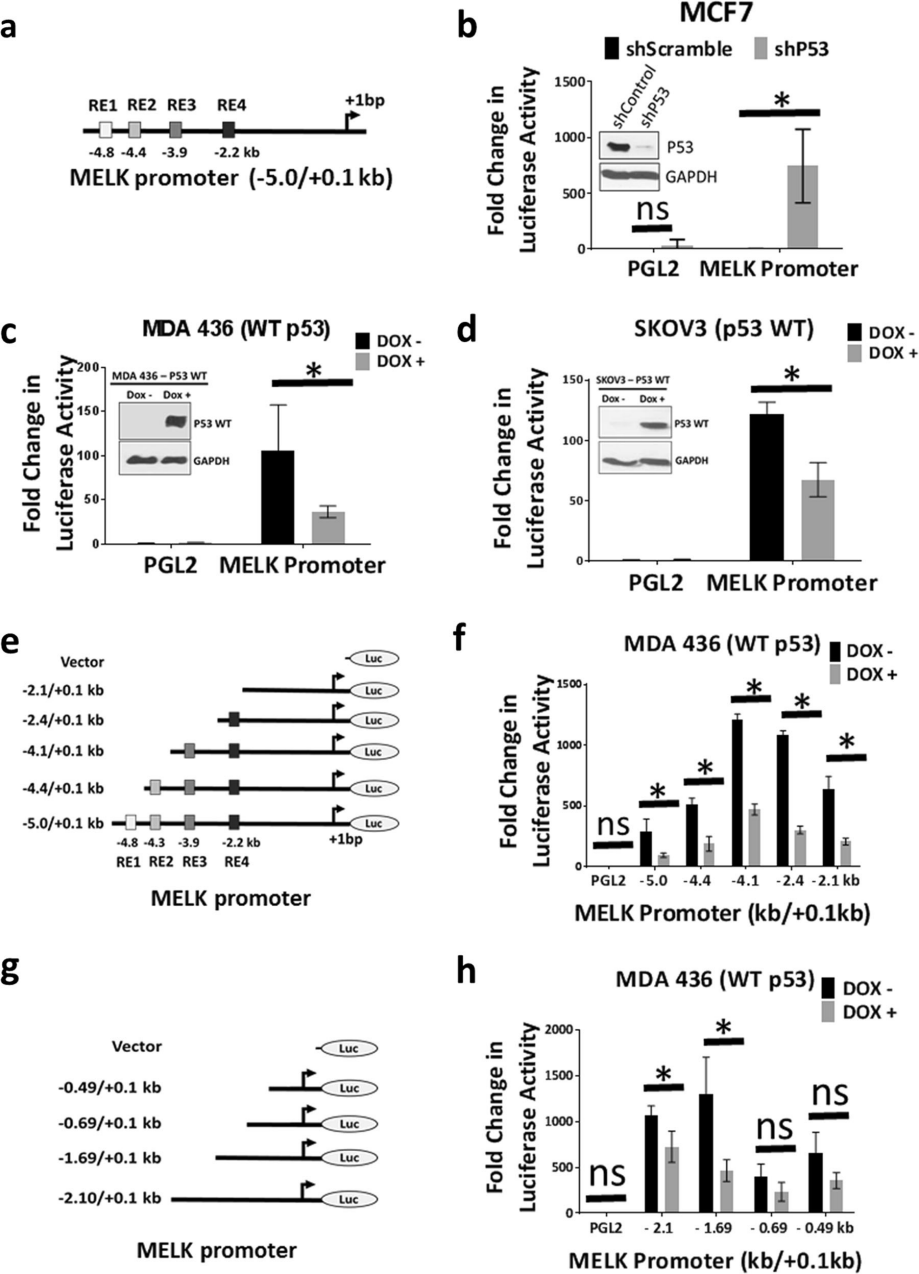
Inhibition of wild-type p53 elevates MELK promoter activity. **a** Schematic diagram of MELK promoter cloned into PGL2-basic luciferase reporter plasmid with predicted p53-binding elements within −5.0 kb. **b** Luciferase assay to determine MELK promoter activity in MCF7 cells after knocking down of wild-type p53 using shRNA. Inset shows the knockdown of p53. **c** Luciferase assay to determine MELK promoter activity in p53-null cells (MDA 436) after inducing wild-type p53 expression using doxycycline-inducible system. Inset shows the induction of p53 protein by doxycycline (Dox) treatment. **d** Luciferase assay to determine MELK promoter activity in p53-null cells (SKOV3) after inducing wild-type p53 expression using doxycycline-inducible system. Inset shows the induction of p53 protein by doxycycline (Dox) treatment. **e** Schematic diagram of MELK promoter constructs. **f** Luciferase assay to determine MELK promoter (shown in **e**) activity in MDA 436 cells after inducing wild-type p53. **g** Schematic diagram of MELK promoter constructs. **h** Luciferase assay to determine MELK promoter (shown in **g**) activity in MDA 436 cells after inducing wild-type p53. For luciferase assays a minimum of three replicates were used. *Statistical significance of *p*-value < 0.05. Error bars represent ± SD.

To test whether WT p53 controls MELK expression by regulating the activity of the MELK promoter, we cloned a −5 kb promoter region of MELK (isolated from human mammary epithelial cells, HMECs) upstream to the transcription start site. Analysis of MELK promoter (using the TransFac software tool) revealed that the −5 kb region of MELK promoter region contains four potential p53 response elements (RE1 through RE4), as shown in Fig. 4a. First, we measured the activity of MELK promoter in p53-deficient cells to test whether p53 status controls MELK promoter activity. For this, we transfected the −5 kb MELK promoter into MCF7 cells that stably expressed p53 short hairpin RNA (shRNA) to knockdown WT p53 or a control shRNA (shScramble). As shown in the Fig. 4b, depletion of WT p53 in MCF7 cells significantly increased MELK promoter activity, suggesting that loss of WT p53 elevates MELK expression. Consistent with this observation, induction of WT p53, using a Dox-inducible system to induce WT p53, suppressed MELK promoter activity in both MDA MB 436 (Fig. 4c) and SKOV3 (Fig. 4d) cells. These results show that WT p53 represses MELK expression by controlling MELK promoter activity, whereas loss of WT p53 elevates MELK expression. Similarly, inhibition of WT p53 by overexpression of dominant-negative p53 mutants in MCF7 cells (WT p53 cells) also significantly increased MELK promoter activity (Supplementary Fig. 4A, B), suggesting that dominant-negative forms of p53 mutants induce MELK expression by inhibiting the repressive effects of WT p53.

### Deletion of putative p53 response elements does not affect repressive effect of WT p53 on MELK promoter activity

To determine whether any of the four p53 response elements (RE1, RE2, RE3, and RE4) is critical for WT p53-mediated inhibition of MELK promoter (as shown in Fig. 4e), we sequentially deleted these elements and transfected these MELK promoter constructs into p53-null cells (MDA MB 436 and SKOV3) that had been stably transfected with a Dox-inducible WT p53 construct. We then measured promoter luciferase activity in the presence and absence of WT p53 (achieved by culturing with or without Dox). Induction of WT p53 repressed the −5 kb MELK promoter activity, as well as the activity of all truncated MELK promoter constructs to levels comparable to the −5 kb MELK promoter in both MDA MB 436 (Fig. 4f) and SKOV3 (Supplementary Fig. 4C) cells. We also measured the activity of these constructs in MCF7 cells with both intact WT p53 and deficient p53. As shown in Supplementary Fig. 4D, the activity of all of these constructs was very low in intact WT p53 cells (shControl), whereas depletion of WT p53 by shP53 significantly elevated the luciferase activity of all constructs. Similarly, inhibition of WT p53 by overexpressing the dominant-negative p53 mutant (R175H) enhanced the activity of MELK promoters in MCF7 cells (Supplementary Fig. 4E). To further investigate whether WT p53 is recruited to any of these putative response elements on MELK promoter, we performed chromatin immunoprecipitation (ChIP) experiments to determine whether WT p53 is recruited to the MELK promoter. As shown in Supplementary Fig. 4F, WT p53 is not recruited to any of the response elements on the MELK promoter. However, WT p53 is recruited to the p21 promoter (used as a positive control). We analyzed several p53-ChIP-seq data sets to identify WT p53-binding sites on MELK promoter. In one of these data sets, WT p53 is reported to be recruited to MELK at the exon1/intron1 region.^46^ Contrary to this observation, we found that WT p53 is not recruited to this site (Supplementary Fig. 5A). Overall, our results suggest that WT p53 represses MELK promoter activity but does not bind to any of the putative p53 response elements.

Next, to identify the region of MELK promoter that is required for WT p53-dependent suppression of MELK expression, we made additional deletions in the MELK promoter and determined whether any of these deletions block the repressive effect of WT p53 on MELK promoter activity. As shown in Fig. 4g, h and Supplementary Fig. 5B, and using the same Dox-inducible system to induce WT p53 in p53-null cells (MDA 436 cells and SKOV3), deletion of the region between − 1.69 kb and − 0.69 kb significantly reduced p53-dependent suppression of MELK promoter activity.

### WT p53 represses MELK expression by repressing FOXM1 expression

**Fig. 5:**
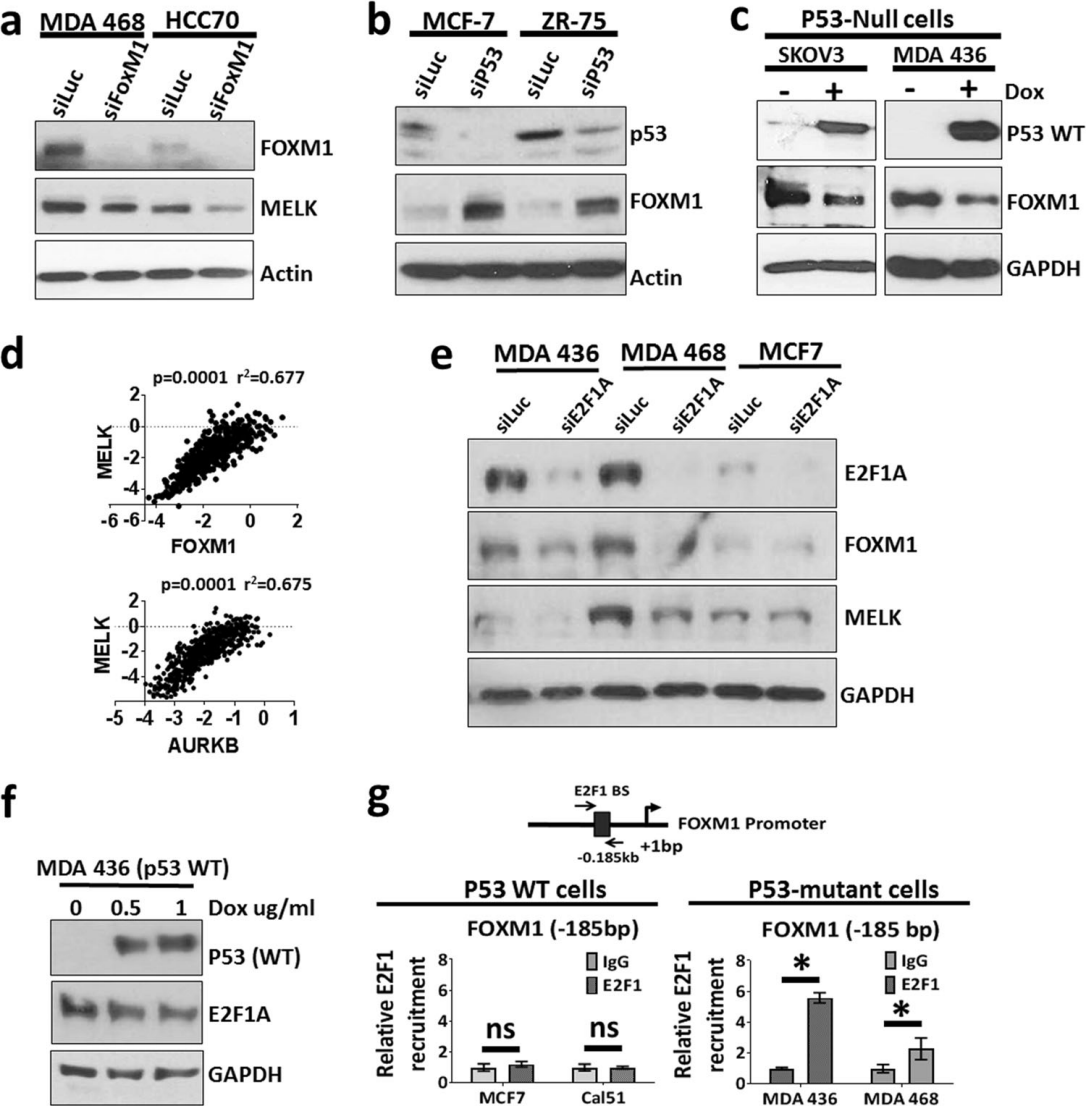
Wild-type p53 represses MELK expression by regulating FOXM1. **a** Western blotting analysis of MELK and FOXM1 protein levels in TNBC cells (MDA 468 and HCC70) after knocking down FOXM1. **b** Western blotting analysis of FOXM1 protein levels after wild-type p53 knockdown in MCF7 and ZR-75 cells. **c** Western blotting analysis of MELK and p53 protein levels in p53-null cells, SKOV3 (left) and MDA MB 436 (right) after inducing wild-type p53 for 48 h. Wild-type p53 was induced, using a doxycycline-inducible system, in p53-null cells. **d** Correlation analysis of MELK mRNA levels with FOXM1 and AURKB (FOXM1 downstream gene) in TCGA breast cancer dataset. The scale for the expression of MELK, FOXM1, and AURKB is log2-median-centered ratio. The Pearson’s correlation *p*-values and *r*^2^-values were calculated using GraphPad prism. **e** Western blotting analysis of MELK, FOXM1, and E2F1 protein levels in p53-mutant cells (MDA 436 and MDA 468) and wild-type p53 cells (MCF7) after E2F1 knocking down. **f** Western blotting analysis of E2F1 in MDA 436 cells after inducing wild-type p53 expression using doxycycline (Dox)-inducible system. **g** E2F1 ChIP assay to determine the relative recruitment of E2F1 to FOXM1 promoter in p53 WT cells (MCF7 and Cal51) and p53 mutant cells (MDA MB 436 and MDA MB 468). Triplicates of ChIP samples were subjected to qPCR assay using primers against FOXM1 promoter (see Supplementary Table 5 for primers). The qPCR data were normalized to % input and then relative recruitment of E2F1 was calculated as a fold change over IgG. *Statistical significance of *p*-value < 0.05. Error bars represent ± SD.

As WT p53 represses MELK promoter activity without binding to its response elements, we hypothesized that WT p53 controls MELK expression by regulating some other transcription factor. We then screened the MELK promoter using TransFac software to identify potential transcription factors that can bind to MELK promoter between − 1.69 and − 0.69 kb. Previously, Wang et al.^24^ reported that FOXM1, an upregulated transcription factor in many human cancers, regulates MELK expression by directly binding to the MELK promoter. Through our TransFac analysis, we identified a novel site for FOXM1 at −686 bp, which is different from the site identified by Wang et al.^24^ (−512 bp). However, consistent with results reported by Wang et al.^23^, knockdown of FOXM1 reduced MELK expression in p53-mutant TNBC cells (Fig. 5a). Knockdown of WT p53 increased FOXM1 expression (Fig. 5b), whereas induction of WT p53 suppressed FOXM1 expression (Fig. 5c), suggesting that WT p53 represses MELK expression by reducing FOXM1 levels. Supporting this data, MELK expression levels correlate with FOXM1 RNA expression levels, as well as with the FOXM1-regulated gene, Aurora kinase B (AURKB), in TCGA breast cancer dataset (Fig. 5d).

Previously, Millour et al.^47^ showed that WT p53 represses FOXM1 levels by reducing E2F1A levels at the FOXM1 promoter. To test whether E2F1 plays a critical role in p53-dependent repression of FOXM1 and MELK, we inhibited E2F1 expression in p53 WT and p53-mutant breast cancer cells. As shown in Fig. 5e, knockdown of E2F1A reduced the expression of FOXM1 and MELK in p53-mutant TNBC cells, whereas knockdown of E2F1A in p53 WT breast cancer cells (MCF7) did not affect FOXM1 and MELK protein levels. We then investigated whether WT p53 represses E2F1A levels to repress FOXM1 and MELK expression. As shown in Fig. 5f, overexpression of WT p53 did not alter the expression levels of E2F1A in p53-null cells (MDA MB 436). However, ChIP analysis revealed that E2F1A is recruited to FOXM1 promoter selectively in p53-mutant cells (MDA MB 436 and MDA MB 468) but not in p53 WT cells (MCF7 and Cal51) (Fig. 5g), suggesting that through an as of yet unknown mechanism, WT p53 regulates the E2F1 activity to repress FOXM1 and MELK expression.

### WT p53 blocks the recruitment of FOXM1 to MELK promoter

**Fig. 6:**
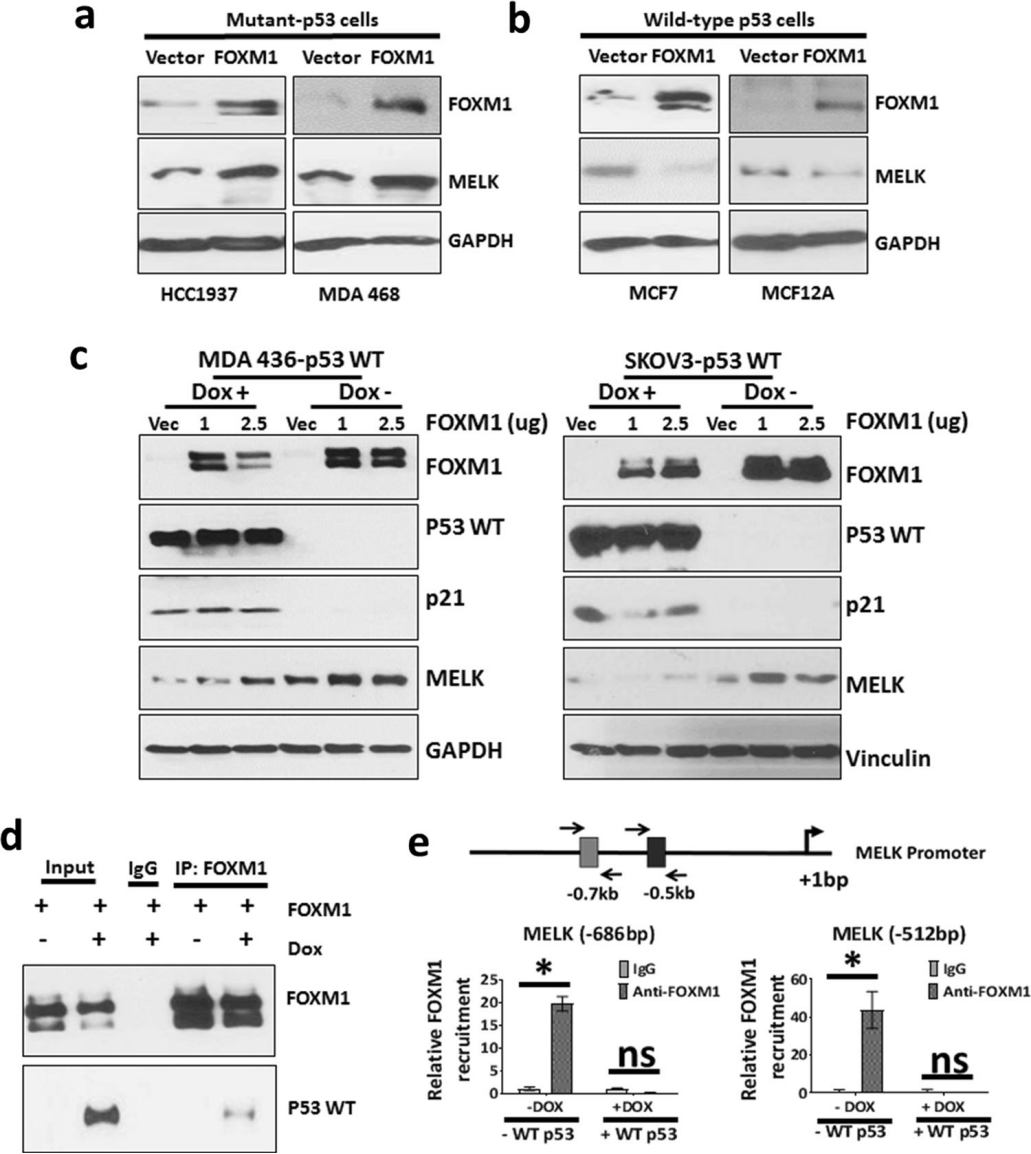
Wild-type p53 represses MELK expression by reducing the recruitment of FOXM1 to MELK promoter. **a** Western blotting analysis of MELK and FOXM1 protein levels in p53-mutant cells (HCC1937 and MDA 468) transfected with FOXM1 cDNA for 48 h. The endogenous FOXM1 band is not seen in vector-transfected cells due to a short western blotting exposure time to clearly show transfected FOXM1 protein. **b** Western blotting analysis of MELK and FOXM1 protein levels in wild-type expressing cells (MCF7 and MCF12A) transfected with FOXM1 cDNA for 48 h. **c** Western blotting analysis of MELK, FOXM1, p21, and p53 in the presence and absence of wild-type p53 in p53-null cells, MDA MB 436 (left) and SKOV3 (right). Wild-type p53 expression was induced using doxycycline-inducible system (Dox treatment). The endogenous FOXM1 band is not seen in vector-transfected cells due to a short western blotting exposure time to clearly show transfected FOXM1 protein. **d** Co-immunoprecipitation assay in MDA MB 436 cells to determine physical interaction between wild-type p53 and FOXM1. Ectopic flag-tagged FOXM1 was transfected into MDA MB 436 cells either treated with no Dox (water alone) or with Dox (in water) to induce wild-type p53. The left two lanes show FOXM1 and p53 proteins in input samples. The middle lane shows the IgG control. The right two lanes show proteins immunoprecipitated by FOXM1 antibody. The last lane shows wild-type p53 co-immunoprecipitated with FOXM1 when FOXM1 is immunoprecipitated. **e** FOXM1 ChIP assay to determine the relative recruitment of FOXM1 to MELK promoter at − 686 bp and − 512 bp in the presence and absence of wild-type p53 (WT p53) in MDA MB 436 cells. FOXM1 and p53 WT were ectopically expressed. The details of the ChIP assay are described in the Materials and Methods section. Triplicates of ChIP samples were subjected to qPCR assay using primers against MELK promoter (see Supplementary Table 5 for primers). The qPCR data were normalized to % input and then relative recruitment of E2F1 was calculated as a fold change over IgG. *Statistical significance of *p*-value < 0.05. Error bars represent ± SD.

To test whether overexpression of FOXM1 can overcome the repressive effects of WT p53 on MELK expression, we overexpressed FOXM1 in WT p53 and p53 mutant cells. As shown in Fig. 6a, b, overexpression of FOXM1 increased MELK expression in p53 mutant (MDA MB 468 and HCC1937) cells, but failed to induce MELK expression in WT p53 cells (MCF7 and MCF12A) (Fig. 6b), suggesting that WT p53 prevents FOXM1’s ability to induce MELK expression. To further test whether p53 blocks FOXM1-induced MELK expression, we ectopically expressed FOXM1 in the presence and absence of WT p53 (using a Dox-inducible system to induce WT p53) in p53-null cells (MDA MB 436 and SKOV3). As shown in the Fig. 6c (in both cell lines), overexpression of FOXM1 increased MELK expression in the absence of WT p53 but did not induce MELK expression in the presence of WT p53. These data suggest that WT p53 represses MELK expression through multiple mechanisms. First, WT p53 causes reduced FOXM1 expression, which in turn leads to reduced MELK expression (Fig. 5c). Second, even in the presence of FOXM1, WT p53 interferes with FOXM1’s ability to induce MELK expression (Fig. 6c). WT p53 causes reduced FOXM1 expression (Fig. 5c) and also inhibits the ability of FOXM1 to induce MELK expression (Fig. 6c). Furthermore, as shown in the Supplementary Fig. 5C, D, overexpression of WT p53 suppresses FOXM1-induced MELK expression and promoter activity. However, it is not clear how WT p53 interferes with FOXM1 ability to induce MELK expression. Previous studies have shown that WT p53 can repress gene expression by blocking the recruitment of transcription factors.^48,49^ Therefore, we hypothesized that WT p53 blocks the recruitment of FOXM1 to the MELK promoter by directly binding to FOXM1. As shown in the Fig. 6d, co-immunoprecitation assay demonstrated that FOXM1 physically interacts with WT p53 in MDA MB 436 cells (last lane in western blotting). To test whether WT p53 blocks the recruitment of FOXM1 to MELK promoter, we performed FOXM1 ChIP experiments. For this study, we ectopically expressed FOXM1 in the absence and presence of WT p53 (induced by Dox) in MDA MB 436 cells. The ChIP study results showed that FOXM1 is recruited to the MELK promoter at both the − 686 bp and − 512 bp sites in the absence of WT p53. However, ectopic expression of WT p53 blocked FOXM1 recruitment to both of these sites in the MELK promoter (Fig. 6e). Ectopic expression of WT p53 also blocked FOXM1 recruitment to the AURKB promoter (Supplementary Fig. 5E), which functions as a positive control for the FOXM1 ChIP assay. These results demonstrate that WT p53 blocks the recruitment of FOXM1 to the MELK promoter, and that when p53 is lost or mutated, FOXM1 is able to bind to the MELK promoter, thus increasing MELK expression.

## Discussion

In this study, we investigated the mechanisms underlying high MELK expression in TNBCs. We discovered that elevated MELK expression is associated with p53 mutation status, and that high MELK expression is caused by loss of WT p53. Our results demonstrate that WT p53 represses MELK expression by inhibiting the expression and promoter-binding ability of FOXM1, a transcription factor that induces MELK transcription. We also showed that mutant p53 induces de-repression of the MELK promoter, thus increasing MELK expression. These results explain the molecular basis for high MELK expression in p53-mutant breast cancers, including TNBCs.

**Fig. 7:**
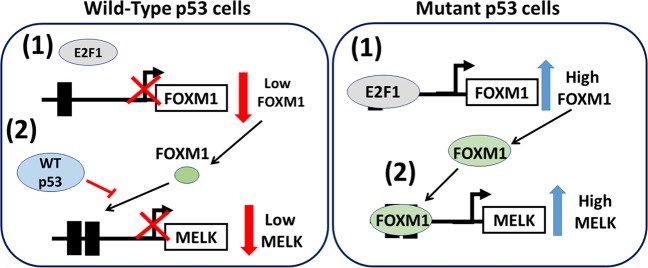
Summary diagram. Summary diagram demonstrating the regulation of MELK by p53, E2F1A, and FOXM1.

Our results are summarized in the model shown in Fig. 7, which shows that in cells containing WT p53, WT p53 actively represses MELK expression by inhibiting FOXM1 transcription and reducing FOXM1 binding to the MELK promoter. However, in cells with mutant p53: (1) E2F1A induces FOXM1 expression and (2) FOXM1 binds to the FOXM1 sites in the MELK promoter, thereby increasing MELK expression.

Previously, we identified MELK, a serine/threonine protein kinase, as a highly expressed kinase in TNBC patients. Several studies reported that high expression of MELK is required for cancer cell growth, invasiveness, cell cycle progression, stemness, radiation-, and chemo-resistance in cancer.^14,17–24,50–55^ Similar to gene knockdown studies, MELK inhibitors also reported to be very effective in both in vitro and in vivo studies despite the fact that these inhibitors have significant off-target effects.^7,56–64^ Recently, Touré et al.^65^ discovered a novel MELK inhibitor using a virtual screening high throughput approach. This compound has highest selectivity for MELK among the MELK inhibitors published to date and inhibited the growth of TNBC cells expressing high MELK but not ER-positive cells that express MELK at low levels.^65^ However, recent studies utilizing gene knockout strategies reported contradicting results that MELK expression is not required for the growth of cancer cells including breast cancer cells, despite the fact that MELK is highly expressed in these cells.^29,66^ Similarly, another study by Huang et al.^30^ reported that the anti-growth effects of small-molecule MELK inhibitors are due to nonspecific targets and increasing the specificity of these inhibitors to MELK reduced their anti-growth effects. However, studies by Wang et al.^31,32^ revealed that knockout of MELK inhibits growth in breast cancer cells under low density culture conditions, whereas knockout of MELK did not affect the growth of ER-positive breast cancer cells in which MELK expression is low.

Our studies demonstrate that high expression of MELK in TNBC cells is due to the loss of WT p53 or mutation in the p53 gene, which is a common phenomenon in 50% of human cancers. p53-mutant cancers have poor outcomes and tend to metastasize frequently, which prompted us to elucidate the role p53 on regulation MELK expression in TNBC. The elevation of MELK expression is also observed in multiple human cancers where p53 is commonly mutated, suggesting that high expression of MELK in p53-mutant tumors is not specific to breast cancer and is a common phenomenon in multiple human cancer types.^3,14,15,18,24,55,67,68^ Thus, our results in TNBCs may also be relevant for other cancer types.

Our results demonstrated that FOXM1 is an important regulator of MELK expression in TNBC cells. FOXM1 is a critical transcription factor, upregulated in many human cancers, including breast cancer.^69,70^ Similar to MELK expression, FOXM1 is highly expressed in p53-mutant cancers due to the loss of WT p53 function. Wang et al.^24^ demonstrated that FOXM1 knockdown reduced MELK, and that FOXM1 binds to the −512 bp site in the MELK promoter. Our studies confirmed the binding of FOXM1 to this site, but also uncovered a novel FOXM1-binding site at -612bp in the MELK promoter. It is not clear whether these two sites function independently or together, stabilizing a multi-protein complex to regulate MELK transcription. A previous study demonstrated that knockout of FOXM1 significantly retards tumor growth and colonization of p53-null lymphoma and sarcoma cells in the lungs.^71^ FOXM1 also has been shown to control the expression of genes to promote cell proliferation, invasiveness, metabolism, and drug resistance.^72–75^

Another study by Barsotti et al.^74^ showed that FOXM1 expression was also suppressed by WT p53 in breast cancer cells. WT p53 represses FOXM1 expression by inhibiting the recruitment of E2F1A^47^ and our studies revealed that knockdown of E2F1A reduces MELK expression by reducing FOXM1 levels in p53-mutant breast cancer cells. E2F1A is a transcription factor that is commonly deregulated in multiple human cancers including breast cancer. High expression of E2F1A is associated with poor prognosis and controls cell cycle, apoptosis, autophagy, differentiation, and stress response in many cancer types.^76–79^ Thus, the results by us and by others demonstrate that the transcriptions factors that control MELK expression, E2F1A, FOXM1, and p53, also regulate key biologic functions of cancer cells.

In summary, we identified that MELK is highly expressed in p53-mutant breast cancers as compared with p53 WT cancers and elucidated the mechanism by which mutant p53 causes upregulation of MELK in these p53-mutant breast cancer cells. Through this study, we discovered that MELK expression is highly upregulated in TNBCs due to the loss of WT p53 activity. We also observed high MELK expression in many other human cancers, where p53 is commonly mutated. Our results show that WT p53 represses MELK expression by inhibiting expression and recruitment of FOXM1 to the MELK promoter. Through these studies, we identified a novel molecular mechanism by which mutant p53 de-represses MELK expression in p53-mutant TNBC breast cancers, and identified FOXM1 and MELK as possible targets for the treatment of p53-mutant breast cancers.

## Methods

### Cell lines and reagents

In this study, we used a panel of cell lines as listed in Supplementary Table 1. All of these cell lines were obtained from ATCC and were grown in the growth medium (listed in Supplementary Table 1). V5-tagged P53 mutant constructs (pLenti-6.0) pInducer20 were obtained from Addgene (listed in Supplementary Table 2). pCMV3 FOXM1 flag-tag was purchased from Sino Biologicals (Cat# HG12392-CF). Three siRNA oligonucleotides for each p53 (3′-GCAUGAACCGGAGGCCCAUTT-5′, 3′-CUACUUCCUGAAAACAACGTT-5′, and 3′-GACUCCAGUGGUAAUCUACTT-5′**)**, E2F1A (Cat# SASI_Hs01_0039325904) and FOXM1 (Cat# SASI_Hs01_00243977, Cat# SASI_Hs01_00052108, and Cat# SASI_Hs01_00193782) were purchased from Sigma-Aldrich.

### Cloning of MELK promoter

For cloning of MELK promoter, we isolated genomic DNA using a DNA extraction kit, (Qiagen, Cat# 69501) from primary HMECs and PCR amplified various lengths of MELK promoter regions using the primer sets listed in Supplementary Table 3. Gel-purified PCR products were then cloned into a linearized PGL2-basic luciferase reporter vector (Promega) using the Quick fusion cloning kit (Biotool, Cat# B2261), according to the manufacturer’s instructions.

### Cloning of WT p53 into pInducer20

Using Gateway method, we cloned WT p53 (pCR8 p53 WT) into pInducer20 (Addgene) following the manufacturer’s instructions. Briefly, 50–150 ng of donor vector (pCR8 p53 WT) and 150 ng of pInducer20 plasmids were mixed with LR Clonase II (Cat# 11791, Invitrogen) and incubated at 25 °C for 1 h and reaction was terminated by adding Proteinase K. The reaction products were then used to transform DH5a bacteria and positive clones were selected on ampicillin containing agar plates^80–84^.

### Luciferase assay

For promoter luciferase assays, we co-transfected 200,000 cells of MDA MB 436 or 100,000 cells of MCF7 and SKOV3 with 300 ng of PGL2 MELK promoter with 50 ng of PhRG *Renilla* vector (internal control) using XTremeGene9 transfection reagent (Cat# XTG9-RO) purchased from Roche. After 48 h, the cells were lysed in 150 μl of passive lysis buffer and 20 μl of clear lysate was used for luciferase activity using a dual-luciferase assay kit (Promega, Cat# E#1910), following the manufacturer’s instructions.

### Transfection and western blotting

To knock down p53, E2F1A, and FOXM1, siRNA transfections were performed using DharmaFect1 transfection reagent (Dharmacon, Cat# T-2001-03), according to the manufacturer’s instructions. To overexpress FOXM1 protein, we transfected breast cells (2 × 10^5^ cells) with FOXM1 plasmid or empty vector in a six-well plate using XtremeGene9 transfection reagent (Cat# XTG9-RO) according to the manufacturer’s instructions. Protein samples were prepared by lysing the cells in RIPA buffer (Sigma-Aldrich, Cat# R0278) supplemented with protease inhibitors and phosphatase inhibitors on ice for 30 min. Lysed cell lysates were collected and centrifuged at 14.000 r.p.m. for 15 min at 4 °C. Equal amount of proteins were subjected to SDS-polyacrylamide gel electrophoresis and western blotting analysis for proteins of interest using antibodies at optimized concentrations. The full list of antibodies used in this study is given in Supplementary Table 4. All western blottings from the same experiment were run in parallel and the full blots are available in Supplementary Fig. 6.

### Generation of stable cells

Stable cells were generated to overexpress p53 (WT and p53 mutants) using lentivirus particles containing p53 constructs. To produce lentivirus, we transfected Lenti-HEK-293 cells with 2 μg of p53 construct, 1.5 μg of viral protein R (VPR), and 0.5 μg of vesicular stomatitis Indiana virus G protein (VSVG), a common coat protein for lentiviral vector expression systems using X-treme-Gene9 transfection reagent (Roche, Cat# XTG9-RO) for 72 h. Viral particles were collected from the medium by collecting supernatant after centrifuging the medium at 4000 r.p.m. for 30 min. One milliliter of lentivirus medium and 10 μg of polybrene were added to infect the cells of interest. After 48 h, G418 (to select WT p53) or blasticidin (to select p53 mutants) were added to select and generate stable cells. Stable cells were tested for expression of WT p53 and p53 mutants through western blotting analysis of HA-tag for p53 WT or V5-tag for p53 mutants.

### ChIP and quantitative reverse-transcriptase PCR analysis

ChIP experiments were performed as described previously with minor modifications. Briefly, for each ChIP, cells were isolated from two 150 cm^2^ plates. Cells were washed with phosphate-buffered saline (PBS) and incubated cells with 1% formalin in PBS for 10 min to crosslink proteins and DNA. Crosslinking was stopped with an incubation with 0.125 M glycine for 5 min. The cells were collected in lysis buffer (1% SDS, 10 mM EDTA in 50 mM Tris, pH 8.1) and sonicated to fragment genomic DNA into 200–500 bp pieces. For each ChIP, 600 μg protein lysate was used to immunoprecipitate p53, E2F1A, or FOXM1. The pulled down DNA fragments were extracted using QIAquick PCR purification kit (Qiagen, cat# 28104) and subjected to quantitative PCR (Q-PCR) using the iTaq SYBR green assay (BioRad, Cat# 1725121). The primer sets used for these assays are listed in Supplementary Table 5. The Q-PCR result was normalized to input fragments and the recruitment of p53, E2F1A, or FOXM1 were presented as a fold change of PCR amplification in p53 or FOXM1 ChIP samples compared with that of IgG samples.

### Analysis of MELK expression and patient survival in breast cancer data sets

To determine the association of MELK expression with p53 mutation status, we obtained MELK mRNA expression data from the Oncomine database. mRNA expression data from Curtis et al.,^38^ Ivshina et al.^39^ (breast cancer data sets), Ding et al.^81^ (lung cancer), Grasso et al.^82^ (prostate cancer), Lindgren et al.^83^ (bladder cancer), and Neale et al.^84^ (brain cancer) were used to compare MELK mRNA levels between p53 WT and p53-mutant cancer patient samples. For breast cancer data sets, we also separated patient samples based on ER status and determined the association between MELK mRNA expression and p53 mutation status. The Student’s *t*-test was used to determine the statistical significance of MELK expression between p53 WT and p53 mutant samples. Three independent data sets (Desmedt, Hatzis, and Schimdt) from the Oncomine database were analyzed to generate metastasis-free survival curves. Samples were dichotomized based on the mean expression level of MELK and log-rank (Mantel–Cox) method and Cox proportional hazards model were used to determine statistical significance. To determine the correlation between MELK expression and p53 regulated genes, we obtained gene expression data from TCGA and Curtis data sets. Correlation curves and Pearson’s *p*-values and *r*^2^-values were generated using GraphPad Prism.

### Reporting summary

Further information on experimental design is available in the Nature Research Reporting Summary linked to this paper.

## Supplementary information

Supplementary Information - Tables, Legends and Figures

Reporting Summary Checklist

## Data Availability

The data generated and analyzed during this study are described in the following metadata record: 10.6084/m9.figshare.11200163.^85^ All mRNA expression data analyzed during the current study were accessed through the Oncomine database (http://www.oncomine.org/) and cBioportal (METABRIC data: https://identifiers.org/cbioportal:brca_metabric, TCGA data: https://identifiers.org/cbioportal:brca_tcga). Survival data were accessed through the Oncomine database. The names of the publicly available datasets (used in this study) as they appear in Oncomine are as follows: TCGA Breast, Curtis Breast, Ivshina Breast, Ma4 Breast, Desmedt Breast, Hatzis Breast, Schmidt Breast, Ding Lung, Grasso Prostate, Lindgren Bladder, and Neale Brain. The raw genomic data of the above Oncomine data sets are also accessible from public repositories. TCGA data are available from the database of Genotypes and Phenotypes (dbGaP) at: https://identifiers.org/dbgap:phs000178.v10.p8. METABRIC data are available from the European Genome-phenome Archive (EGA) at: https://identifiers.org/ega.study:EGAS00000000083. Ivshina Breast, Ma4 Breast, Desmedt Breast, Hatzis Breast, Schmidt Breast, Ding Lung, Grasso Prostate, and Lindgren Bladder are all available from the Gene Expression Omnibus (GEO) repository at https://identifiers.org/geo:GSE4922, https://identifiers.org/geo:GSE14548, https://identifiers.org/geo:GSE7390, https://identifiers.org/geo:GSE25066, https://identifiers.org/geo:GSE11121, https://identifiers.org/geo:GSE12667, https://identifiers.org/geo:GSE35988, and https://identifiers.org/geo:GSE19915, respectively. Neale Brain data are available from dbGaP at: https://identifiers.org/dbgap:phs000469.v7.p1. The data sets generated during the study will be made available on request from the corresponding author Dr Powel H. Brown, as described in the figshare metadata record above. Uncropped blots are available as part of supplementary information (Supplementary Fig. 6).

## References

[1] Speers C (2009). Identification of novel kinase targets for the treatment of estrogen receptor-negative breast cancer. Clin. Cancer Res..

[2] Pitner MK, Taliaferro JM, Dalby KN, Bartholomeusz C (2017). MELK: a potential novel therapeutic target for TNBC and other aggressive malignancies. Expert Opin. Ther. Targets.

[3] Ganguly R, Hong CS, Smith LG, Kornblum HI, Nakano I (2014). Maternal embryonic leucine zipper kinase: key kinase for stem cell phenotype in glioma and other cancers. Mol. Cancer Ther..

[4] Ganguly R (2015). MELK-a conserved kinase: functions, signaling, cancer and controversy. Clin. Transl. Med..

[5] Pickard MR (2009). Dysregulated expression of Fau and MELK is associated with poor prognosis in breast cancer. Breast Cancer Res..

[6] Li S (2016). Maternal embryonic leucine zipper kinase serves as a poor prognosis marker and therapeutic target in gastric cancer. Oncotarget.

[7] Kohler RS (2017). MELK expression in ovarian cancer correlates with poor outcome and its inhibition by OTSSP167 abrogates proliferation and viability of ovarian cancer cells. Gynecol. Oncol..

[8] Sun X, Gao L, Chien HY, Li WC, Zhao J (2013). The regulation and function of the NUAK family. J. Mol. Endocrinol..

[9] Heyer BS, Kochanowski H, Solter D (1999). Expression of Melk, a new protein kinase, during early mouse development. Dev. Dyn..

[10] Heyer BS, Warsowe J, Solter D, Knowles BB, Ackerman SL (1997). New member of the Snf1/AMPK kinase family, Melk, is expressed in the mouse egg and preimplantation embryo. Mol. Reprod. Dev..

[11] Tian S (2010). Biological functions of the genes in the mammaprint breast cancer profile reflect the hallmarks of cancer. Biomark. Insights.

[12] van ‘t Veer, L. J. et al. Gene expression profiling predicts clinical outcome of breast cancer. *Nature* **415**, 530–536 (2002). 10.1038/415530a 11823860

[13] Parker JS (2009). Supervised risk predictor of breast cancer based on intrinsic subtypes. J. Clin. Oncol..

[14] Hebbard LW (2010). Maternal embryonic leucine zipper kinase is upregulated and required in mammary tumor-initiating cells in vivo. Cancer Res..

[15] Joshi K (2013). MELK-dependent FOXM1 phosphorylation is essential for proliferation of glioma stem cells. Stem Cells (Dayt., Ohio).

[16] Kim SH (2015). EZH2 protects glioma stem cells from radiation-induced cell death in a MELK/FOXM1-dependent manner. Stem Cell Rep..

[17] Badouel C, Chartrain I, Blot J, Tassan JP (2010). Maternal embryonic leucine zipper kinase is stabilized in mitosis by phosphorylation and is partially degraded upon mitotic exit. Exp. Cell Res..

[18] Choi S, Ku JL (2011). Resistance of colorectal cancer cells to radiation and 5-FU is associated with MELK expression. Biochem. Biophys. Res. Commun..

[19] Du T (2014). Maternal embryonic leucine zipper kinase enhances gastric cancer progression via the FAK/Paxillin pathway. Mol. Cancer.

[20] Moreno CS (2016). MELK kinase holds promise as a new radiosensitizing target and biomarker in triple-negative breast cancer. J. Thorac. Dis..

[21] Nakano I (2008). Maternal embryonic leucine zipper kinase is a key regulator of the proliferation of malignant brain tumors, including brain tumor stem cells. J. Neurosci. Res..

[22] Speers C (2016). Maternal embryonic leucine zipper kinase (MELK) as a novel mediator and biomarker of radioresistance in human breast cancer. Clin. Cancer Res..

[23] Wang Y (2016). Mitotic MELK-eIF4B signaling controls protein synthesis and tumor cell survival. Proc. Natl Acad. Sci. USA.

[24] Wang Y (2014). MELK is an oncogenic kinase essential for mitotic progression in basal-like breast cancer cells. eLife.

[25] Lin ML, Park JH, Nishidate T, Nakamura Y, Katagiri T (2007). Involvement of maternal embryonic leucine zipper kinase (MELK) in mammary carcinogenesis through interaction with Bcl-G, a pro-apoptotic member of the Bcl-2 family. Breast Cancer Res..

[26] Chlenski Alexandre, Park Chanyoung, Dobratic Marija, Salwen Helen R., Budke Brian, Park Jae-Hyun, Miller Ryan, Applebaum Mark A., Wilkinson Emma, Nakamura Yusuke, Connell Philip P., Cohn Susan L. (2019). Maternal Embryonic Leucine Zipper Kinase (MELK), a Potential Therapeutic Target for Neuroblastoma. Molecular Cancer Therapeutics.

[27] Meel MH (2018). MELK inhibition in diffuse intrinsic pontine glioma. Clin. Cancer Res..

[28] Guan S (2018). MELK is a novel therapeutic target in high-risk neuroblastoma. Oncotarget.

[29] Lin, A., Giuliano, C. J., Sayles, N. M. & Sheltzer, J. M. CRISPR/Cas9 mutagenesis invalidates a putative cancer dependency targeted in on-going clinical trials. *eLife* **6**, 10.7554/eLife.24179 (2017). 10.7554/eLife.24179 PMC5365317 28337968

[30] Huang, H. T. et al. MELK is not necessary for the proliferation of basal-like breast cancer cells. *eLife* **6**, 10.7554/eLife.26693 (2017). 10.7554/eLife.26693 PMC5605198 28926338

[31] Wang Y, Li BB, Li J, Roberts TM, Zhao JJ (2018). A conditional Dependency on MELK for the proliferation of triple-negative breast cancer cells. iScience.

[32] Wang, Y. et al. Correction: MELK is an oncogenic kinase essential for mitotic progression in basal-like breast cancer cells. *eLife* **7**10.7554/eLife.36414 (2018). 10.7554/eLife.36414 PMC5847332 29528283

[33] Riley T, Sontag E, Chen P, Levine A (2008). Transcriptional control of human p53-regulated genes. Nat. Rev. Mol. Cell Biol..

[34] Vousden KH, Prives C (2009). Blinded by the light: the growing complexity of p53. Cell.

[35] Muller PA, Vousden KH (2013). p53 mutations in cancer. Nat. Cell Biol..

[36] Petitjean A (2007). Impact of mutant p53 functional properties on TP53 mutation patterns and tumor phenotype: lessons from recent developments in the IARC TP53 database. Hum. Mutat..

[37] Pereira B (2016). The somatic mutation profiles of 2,433 breast cancers refines their genomic and transcriptomic landscapes. Nat. Commun..

[38] Curtis C (2012). The genomic and transcriptomic architecture of 2,000 breast tumours reveals novel subgroups. Nature.

[39] Ivshina AV (2006). Genetic reclassification of histologic grade delineates new clinical subtypes of breast cancer. Cancer Res..

[40] Minn AJ (2005). Genes that mediate breast cancer metastasis to lung. Nature.

[41] Desmedt C (2007). Strong time dependence of the 76-gene prognostic signature for node-negative breast cancer patients in the TRANSBIG multicenter independent validation series. Clin. Cancer Res..

[42] Esserman LJ (2012). Chemotherapy response and recurrence-free survival in neoadjuvant breast cancer depends on biomarker profiles: results from the I-SPY 1 TRIAL (CALGB 150007/150012; ACRIN 6657). Breast Cancer Res. Treat..

[43] Schmidt M (2008). The humoral immune system has a key prognostic impact in node-negative breast cancer. Cancer Res..

[44] Powell E, Piwnica-Worms D, Piwnica-Worms H (2014). Contribution of p53 to metastasis. Cancer Discov..

[45] Fischer M (2017). Census and evaluation of p53 target genes. Oncogene.

[46] Menendez D (2013). Diverse stresses dramatically alter genome-wide p53 binding and transactivation landscape in human cancer cells. Nucleic Acids Res..

[47] Millour J (2011). ATM and p53 regulate FOXM1 expression via E2F in breast cancer epirubicin treatment and resistance. Mol. Cancer Ther..

[48] Dalvai M, Mondesert O, Bourdon JC, Ducommun B, Dozier C (2011). Cdc25B is negatively regulated by p53 through Sp1 and NF-Y transcription factors. Oncogene.

[49] Hwang CI (2011). Wild-type p53 controls cell motility and invasion by dual regulation of MET expression. Proc. Natl Acad. Sci. USA.

[50] Gray D (2005). Maternal embryonic leucine zipper kinase/murine protein serine-threonine kinase 38 is a promising therapeutic target for multiple cancers. Cancer Res..

[51] Hiwatashi K (2016). Expression of maternal embryonic leucine zipper kinase (MELK) correlates to malignant potentials in hepatocellular carcinoma. Anticancer Res..

[52] Jiang P, Zhang D (2013). Maternal embryonic leucine zipper kinase (MELK): a novel regulator in cell cycle control, embryonic development, and cancer. Int. J. Mol. Sci..

[53] Liu H (2017). MELK and EZH2 cooperate to regulate medulloblastoma cancer stem-like cell proliferation and differentiation. Mol. Cancer Res..

[54] Vulsteke V (2004). Inhibition of spliceosome assembly by the cell cycle-regulated protein kinase MELK and involvement of splicing factor NIPP1. J. Biol. Chem..

[55] Xia H (2016). MELK is an oncogenic kinase essential for early hepatocellular carcinoma recurrence. Cancer Lett..

[56] Alachkar H (2014). Preclinical efficacy of maternal embryonic leucine-zipper kinase (MELK) inhibition in acute myeloid leukemia. Oncotarget.

[57] Beke, L. et al. MELK-T1, a small-molecule inhibitor of protein kinase MELK, decreases DNA-damage tolerance in proliferating cancer cells. *Biosci. Rep.* **35**, 10.1042/bsr20150194 (2015). 10.1042/BSR20150194 PMC4643329 26431963

[58] Cho YS, Kang Y, Kim K, Cha YJ, Cho HS (2014). The crystal structure of MPK38 in complex with OTSSP167, an orally administrative MELK selective inhibitor. Biochem. Biophys. Res. Commun..

[59] Chung S (2016). Preclinical evaluation of biomarkers associated with antitumor activity of MELK inhibitor. Oncotarget.

[60] Chung S, Nakamura Y (2013). MELK inhibitor, novel molecular targeted therapeutics for human cancer stem cells. Cell Cycle (Georget., Tex.).

[61] Chung S (2012). Development of an orally-administrative MELK-targeting inhibitor that suppresses the growth of various types of human cancer. Oncotarget.

[62] Edupuganti R (2017). Discovery of a potent inhibitor of MELK that inhibits expression of the anti-apoptotic protein Mcl-1 and TNBC cell growth. Bioorg. Med. Chem..

[63] Ji W (2016). OTSSP167 abrogates mitotic checkpoint through inhibiting multiple mitotic kinases. PLoS ONE.

[64] Stefka AT (2016). Anti-myeloma activity of MELK inhibitor OTS167: effects on drug-resistant myeloma cells and putative myeloma stem cell replenishment of malignant plasma cells. Blood Cancer J..

[65] Toure BB (2016). Toward the validation of maternal embryonic leucine zipper kinase: discovery, optimization of highly potent and selective inhibitors, and preliminary biology insight. J. Med. Chem..

[66] Giuliano, C. J., Lin, A., Smith, J. C., Palladino, A. C. & Sheltzer, J. M. MELK expression correlates with tumor mitotic activity but is not required for cancer growth. *eLife* **7**, 10.7554/eLife.32838 (2018). 10.7554/eLife.32838 PMC5805410 29417930

[67] Calcagno DQ (2016). Identification of IL11RA and MELK amplification in gastric cancer by comprehensive genomic profiling of gastric cancer cell lines. World J. Gastroenterol..

[68] Goto Y (2017). Impact of novel miR-145-3p regulatory networks on survival in patients with castration-resistant prostate cancer. Br. J. Cancer.

[69] Koo CY, Muir KW, Lam EW (2012). FOXM1: from cancer initiation to progression and treatment. Biochim. Biophys. Acta.

[70] Myatt SS, Lam EW (2008). Targeting FOXM1. Nat. Rev. Cancer.

[71] Wang Z (2013). Targeting FoxM1 effectively retards p53-null lymphoma and sarcoma. Mol. Cancer Ther..

[72] Raychaudhuri P, Park HJ (2011). FoxM1: a master regulator of tumor metastasis. Cancer Res..

[73] Halasi M, Gartel AL (2012). Suppression of FOXM1 sensitizes human cancer cells to cell death induced by DNA-damage. PLoS ONE.

[74] Barsotti AM, Prives C (2009). Pro-proliferative FoxM1 is a target of p53-mediated repression. Oncogene.

[75] Wierstra I, Alves J (2007). FOXM1, a typical proliferation-associated transcription factor. Biol. Chem..

[76] Chen HZ, Tsai SY, Leone G (2009). Emerging roles of E2Fs in cancer: an exit from cell cycle control. Nat. Rev. Cancer.

[77] Hollern DP, Honeysett J, Cardiff RD, Andrechek ER (2014). The E2F transcription factors regulate tumor development and metastasis in a mouse model of metastatic breast cancer. Mol. Cell. Biol..

[78] Li Y (2018). Expression patterns of E2F transcription factors and their potential prognostic roles in breast cancer. Oncol. Lett..

[79] Meng P, Ghosh R (2014). Transcription addiction: can we garner the Yin and Yang functions of E2F1 for cancer therapy?. Cell Death Dis..

[80] Ma XJ, Dahiya S, Richardson E, Erlander M, Sgroi DC (2009). Gene expression profiling of the tumor microenvironment during breast cancer progression. Breast Cancer Res..

[81] Ding L (2008). Somatic mutations affect key pathways in lung adenocarcinoma. Nature.

[82] Grasso CS (2012). The mutational landscape of lethal castration-resistant prostate cancer. Nature.

[83] Lindgren D (2006). Molecular characterization of early-stage bladder carcinomas by expression profiles, FGFR3 mutation status, and loss of 9q. Oncogene.

[84] Neale G (2008). Molecular characterization of the pediatric preclinical testing panel. Clin. Cancer Res..

[85] Reddy, B. L. et al. Metadata supporting data files in the published article: Mutant P53 induces MELK expression by release of wild-type P53-dependent suppression of FOXM1. *figshare*. 10.6084/m9.figshare.11200163 (2019).

