# Supplementary material for: Mutant P53 induces MELK expression by release of wild-type P53-dependent suppression of FOXM1
Source: NPJ Breast Cancer. 2020 Jan 3;6:2. doi: 10.1038/s41523-019-0143-5 (PMC6941974; doi:10.1038/s41523-019-0143-5)

**Supplementary Table 1:** List of cell lines and their status.

| **Cell line** | **Cancer type** | **P53 status** | **Growth medium** |
|---|---|---|---|
| MCF12A | Non-cancerous | Wild-type | DMEM/F12 |
| hMEC-hTert | Non-cancerous | Wild-type | MEGM |
| MCF-7 | ER-postive | Wild-type | DMEM |
| ZR-75 | ER-positive | Wild-type | DMEM |
| DU4475 | Triple-Negative | Wild-type | DMEM |
| MDA MB 175VII | Triple-Negative | Wild-type | DMEM |
| Cal51 | Triple-Negative | Wild-type | DMEM |
| MDA MB 231 | Triple-Negative | R280K | DMEM |
| MDA MB 468 | Triple-Negative | R273L | DMEM |
| MDA MB 436 | Triple-Negative | E204Fs (Null) | DMEM |
| HCC 1143 | Triple-Negative | R248Q | RPMI |
| HCC 70 | Triple-Negative | R248Q | RPMI |
| HCC 38 | Triple-Negative | R273L | RPMI |
| HCC 1954 | Her2-Positve | Y163S | RPMI |
| SKOV3 | Ovarian cancer | P89fs (Null) | McKoy |
| H1299 | Lung cancer | Null | RPMI |
| Lenti-HEK-293 | Non-cancerous | Wild-type | DMEM |

**Supplementary Table 2:** List of plasmids.

| Plasmid | Source | Catalog # |
| --- | --- | --- |
| Pleni6 V5 GFP | Addgene | 40125 |
| pLenti6/V5-p53_R175H | Addgene | 22936 |
| pLenti6/V5-p53_R249S | Addgene | 22935 |
| pLenti6/V5-p53_R273H | Addgene | 22934 |
| pLenti6/V5-p53_R280K | Addgene | 22933 |
| pInducer20 | Addgene | 44012 |
| pCMV3-FOXM1-flag | Sino Biological | HG12392-CF |

**Supplementary Table 3:** MELK promoter cloning primers.

| Promoter (Kb) | Forward primer |
|---|---|
| -5.0/+0.1 kb | 5’-AGGTACCGAGCTCTTAACCCAGGTGGTGGAGGTT-3’ |
| -4.4/+0.1 kb | 5’-AGGTACCGAGCTCTTA CTCTTTTTGTCCAGGCTGGA-3’ |
| -4.0/+0.1 kb | 5’-AGGTACCGAGCTCTTATCTCTTTCCCTCTCCTCAGG-3’ |
| -2.4/+0.1 kb | 5’-AGGTACCGAGCTCTTAATGGAGATGTACAAGGAAATGAATG-3’ |
| -2.1/+0.1 kb | 5’-AGGTACCGAGCTCTTA GAGAATGGCGTGAACCCA-3’ |
| -1.79/+0.1 kb | 5’-AGGTACCGAGCTCTTAGATGGAAGCTCCTCTTGGTG-3’ |
| -0.67/+0.1 kb | 5’-AGGTACCGAGCTCTTATTTGGTTCCAGGGATTACTGG-3’ |
| -0.49/+0.1 kb | 5’-AGGTACCGAGCTCTTAAGCCTGGCCAACATAGCG-3’ |
| Reverse primer | 5’-CTTACTTAGATCTCGAGGGGCCTGCTGTCCTGAGA-3’ |

**Supplementary Table 4:** List of Antibodies and source.

| Antibody | Assay | Source | Catalog # | Dilution |
|---|---|---|---|---|
| MELK | Western blot | R&D | AF4820 | 1:5000 |
| FOXM1 | Western blot | Santa Cruz biotechnology | Sc-502 | 1:4000 |
| FOXM1 | ChIP | Santa Cruz biotechnology | sc-376471X | 2ug/IP |
| P53 | Western blot | Santa Cruz biotechnology | Sc-126 | 1:6000 |
| P53 | ChIP | Santa Cruz biotechnology | Sc-126X | 2ug/IP |
| HA | Western blot | Sigma Aldrich | H9658 | 1:6000 |
| V5 | Western blot | Cell signaling | 13202 | 1:6000 |
| GAPDH | Western blot | Sigma Aldrich | G8795 | 1:10000 |
| Vinculin | Western blot | Sigma Aldrich | SAB4200080 | 1:10000 |
| Actin | Western blot | Sigma Aldrich | A4700 | 1:10000 |
| P21 | Western blot | Cell signaling | 2947 | 1:2000 |
| E2F1 | ChIP & Western Blot | Santa Cruz Biotechnology | Sc-251 | 1:2000 |

**Supplementary Table 5:** Primer list for ChIP assays.

| Primer | Assay | Sequence |
|---|---|---|
| RE1 forward | P53 ChIP | 5’-GGGAGGCTGAGGCAGGTA-3’ |
| RE1 Reverse | P53 ChIP | 5’-TAGGGATAGGGTTTCACCATATTG-3’ |
| RE2 Forward | P53 ChIP | 5’-ACTTTGGGAGGCTGAGATGG-3’ |
| RE2 Reverse | P53 ChIP | 5’-ATGGCCTGACTGGGTAGGT-3’ |
| RE3 Forward | P53 ChIP | 5’-CAAAGGACAGGTTGACTCTCTTG-3’ |
| RE3 Reverse | P53 ChIP | 5’-TAATTACGTCACCGCATTCCA-3’ |
| RE4 Forward | P53 ChIP | 5’-AAATTAGCCGGGCATGGT-3’ |
| RE4 Reverse | P53 ChIP | 5’-TATTCAGGCTTCACTTCCAATTC-3’ |
| P21 Forward | P53 ChIP | 5’-GCTCCCTCATGGGCAAACTCACT- 3’ |
| P21 Reverse | P53 ChIP | 5’-TGGCTGGTCTACCTGGCTCCTCT- 3’ |
| MELK (-686) Forward | FOXM1 ChIP | 5’-AACCAATCCAGCTGTTTCCA-3’ |
| MELK (-686) Reverse | FOXM1 ChIP | 5’-CCAGTAATCCCTGGAACCAA-3’ |
| MELK (-512) Forward | FOXM1 ChIP | 5'-AGGCTGAGGCGGGAGGATCGCTT-3' |
| MELK (-512) Reverse | FOXM1 ChIP | 5'-GTGTTGCCACGAGGAATAAGAACC-3' |
| AURKB Forward | FOXM1 ChIP | 5’-TCT AAC TTC TCT GCC CGA TGG AG-3’ |
| AURKB Reverse | FOXM1 ChIP | 5’-GCA ACG AAA GGT CTA TTG GTG G-3’ |
| Foxm1 (-185) Forward | E2F1 ChIP | 5’-CCACTTCTTCCCCCACAAG-3’ |
| Foxm1 (-185) Reverse | E2F1 ChIP | 5’-CCGGAGCTTTCAGTTTGTTC-3’ |

**Supplementary Figure Legends:**

**Supplementary Figure 1.** Analysis of MELK mRNA expression levels in TNBCs compared to normal breast and non-TNBC tumors in both Curtis **(A)** and TCGA **(B)** datasets. Analysis of MELK mRNA expression in normal breast, early stage (DCIS) of breast cancer and invasive cancer in Ma-4 breast dataset (matched tissues) and Curtis dataset (**C** and **D**) [38,80]. The scale for MELK expression is log2 median-centered ratio. Error bars represents a range of minimum to maximum log2-median centered MELK expression levels. The box encompasses the upper and lower quartiles. The central line represents the median. **E.** Kaplan-Meier curves of metastasis-free survival of all breast cancer patients in 3 breast cancer datasets stratified by MELK expression. n=198 in Desmedt, n=508 in Hatzis, n=200 in Schmidt dataset for both MELK-Low and MELK-High respectively. * Indicates statistical significance of p-value <0.05. Error bars represent +/- SEM.

**Supplementary Figure 2. Correlation between MELK expression and p53-mutation status. A.** Comparison of MELK expression in matched normal breast and mammary tumor. Elevation of MELK expression in p53-mutant breast cancer patients (TCGA, Provisional dataset) is higher than p53 wild-type breast cancers. **B.** Comparison of MELK expression between p53 wild-type and p53-mutant breast cancer samples in both ER-positive and ER-negative breast cancer data sets (Ivshina and Curtis). **C.** Comparison of MELK expression between p53 wild-type and p53-mutant human cancers [81-84]. The scale for MELK expression is log2 median-centered ratio. * Indicates statistical significance of p-value <0.05. Error bars represents a range of minimum to maximum log2-median centered MELK expression levels. The box encompasses the upper and lower quartiles. The central line represents the median.

**Supplementary Figure 3. A.** Western blotting analysis of MELK and p53 protein levels in p53-null cells, MDA MB 436, after inducing wild-type p53 for 48 hours. Wild-type p53 was induced by treating with doxycycline (Dox) in a dose dependent manner. Non-specific effects of doxycycline on MELK protein levels were determined by treating vector expressing MDA 436 cells with doxycycline. **B.** Western blotting analysis of MELK and p53 protein levels in p53-null cells, MDA MB 436, after inducing wild-type p53 for 48 hours. Inhibitors of protease-mediated protein degradation were added for 8 hours at the end of experiment to determine the effect of wild-type p53 on MELK protein degradation. Correlation of MELK mRNA levels with wild-type p53-repressed and wild-type p53-induced genes breast cancer datasets (**C&D**).

**Supplementary Figure 4. Identification of wild-type p53 response region in within -5.0kb MELK promoter. A.** Western blotting analysis of p53 and GAPDH in MCF-7 cells stably expressing dominant-negative p53-mutants. **B.** Luciferase assay to determine MELK-promoter activity in MCF-7 cells expressing p53-mutants as shown in panel **A**. **C.** Luciferase assay to determine MELK-promoter constructs (shown in **Figure 4E**) activity in SKOV3 cells after inducing wild-type p53. **D.** Analysis of MELK promoter constructs (shown in **Figure 4E**) in p53 depleted cells (MCF-7). **E.** Analysis of MELK promoter constructs activity in MCF-7 cells that are stably expressing p53-mutant (p53 R175H). **F.** Wild-p53 ChIP assay to determine the relative recruitment of wild-type p53 to MELK promoter at predicted response elements (RE1, RE2, RE3 & RE4). p53 recruitment to p21 promoter region was used as a positive control for p53 ChIP assay. The qPCR data were normalized to % input and then relative recruitment of p53 was calculated as a fold change over IgG as described in the Materials and Methods section. * Indicates statistical significance of p-value <0.05. Error bars represent +/- SD.

**Supplementary Figure 5. Wild-type p53 blocks FOXM1-induced MELK promoter activity. A.** Wild-p53 ChIP assay to determine the relative recruitment of wild-type p53 to MELK at first

exon/intronic region. **B.** Luciferase assay to determine MELK-promoter constructs (shown in panel **4G**) activity in SKOV3 cells after inducing wild-type p53. **C.** Western blotting analysis of p53 (Wild-type), MELK and FOXM1. FOXM1 protein is not seen in vector-transfected cells due to a short western blot exposure time to clearly show transfected FOXM1 protein. 1ug of FOXM1 was ectopically expressed in to SKOV3 in the absence and presence of wild-type p53. Wild-type p53 expression was induced in SKOV3 cells (doxycycline-inducible p53 system) by treating doxycycline (Dox). **D.** Luciferase assay to determine MELK promoter activity in p53-null cells (SKOV3) in the presence and absence of FOXM1 and wild-type p53 (as shown in panel **C**). Three MELK constructs of 5.0 kb, 4.4 kb, and 4.1 kb were used in this assay. **E.** FOXM1 ChIP assay to determine the relative recruitment of FOXM1 to Aurora kinase B (AURKB) promoter in the presence and absence of wild-type p53 (WT p53) in MDA MB 436 cells. The qPCR data were normalized to % input and then relative recruitment of FOXM1 was calculated as a fold change over IgG. * Indicates statistical significance of p-value <0.05. Error bars represent +/- SD.

**Supplementary Figure 6.** Raw data for Western blots for each figure. The raw data for the Western blots included in each figure and supplementary figure is provided sequentially.

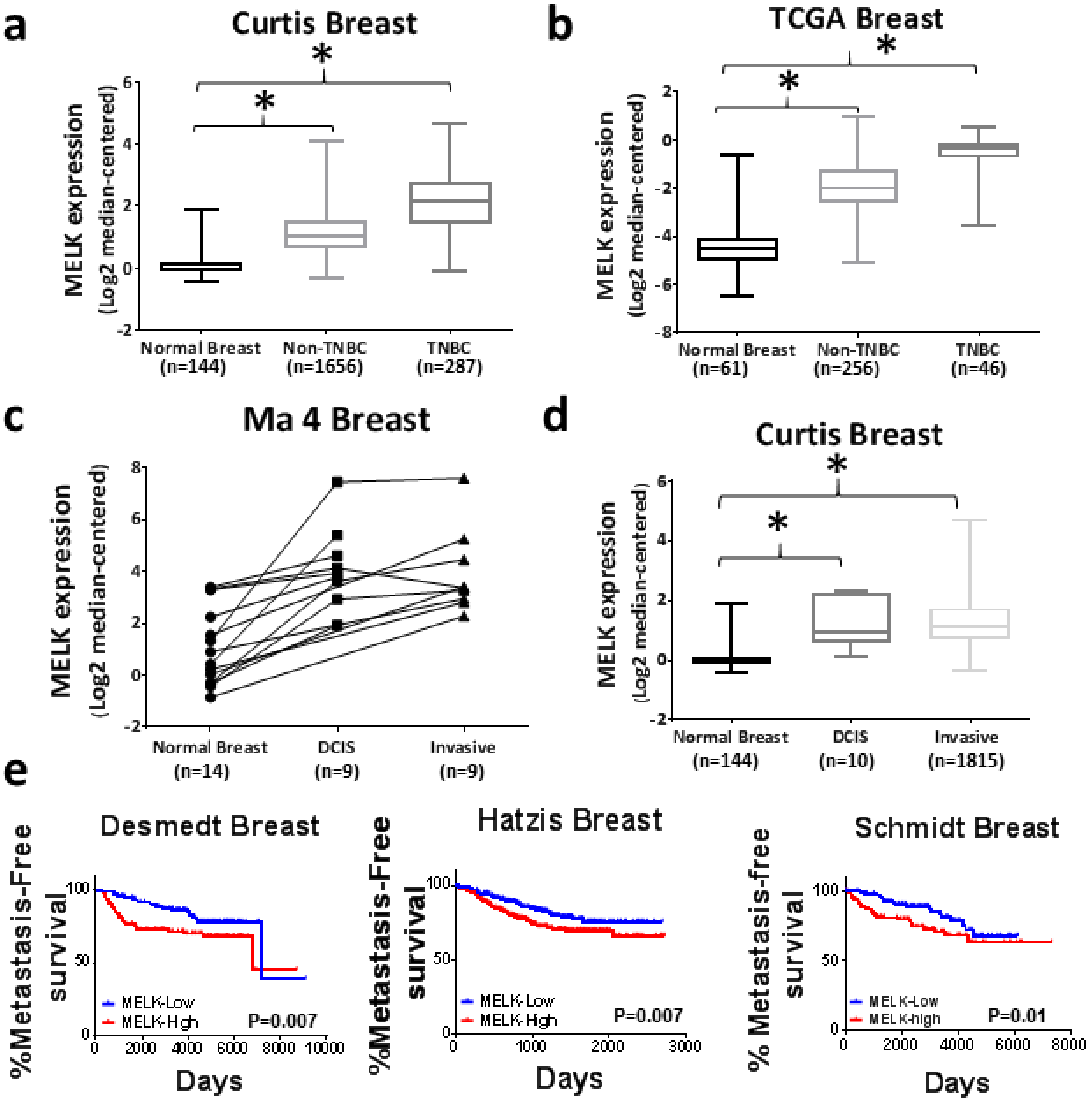


**Supplementary Figure 1**

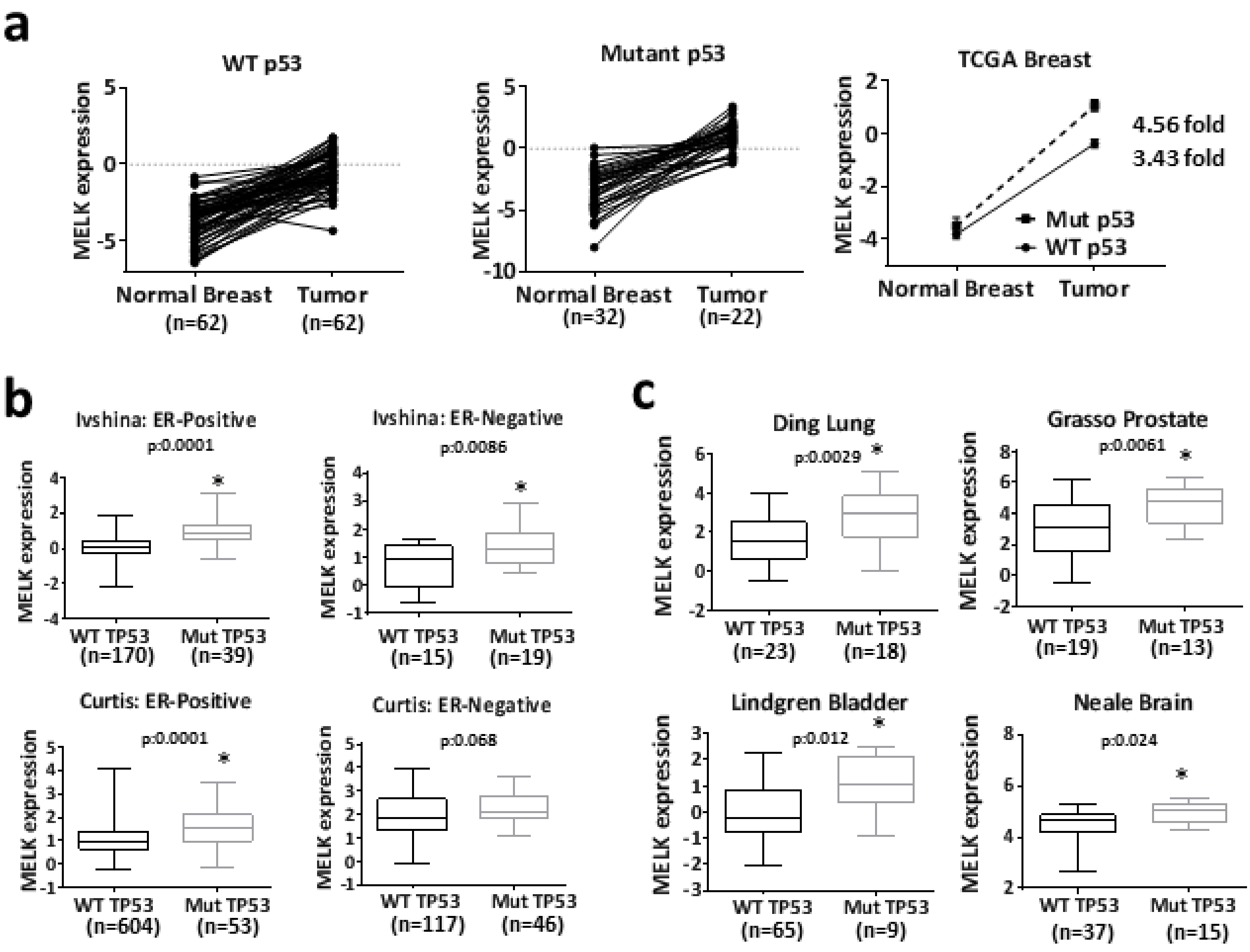


**Supplementary Figure 2**

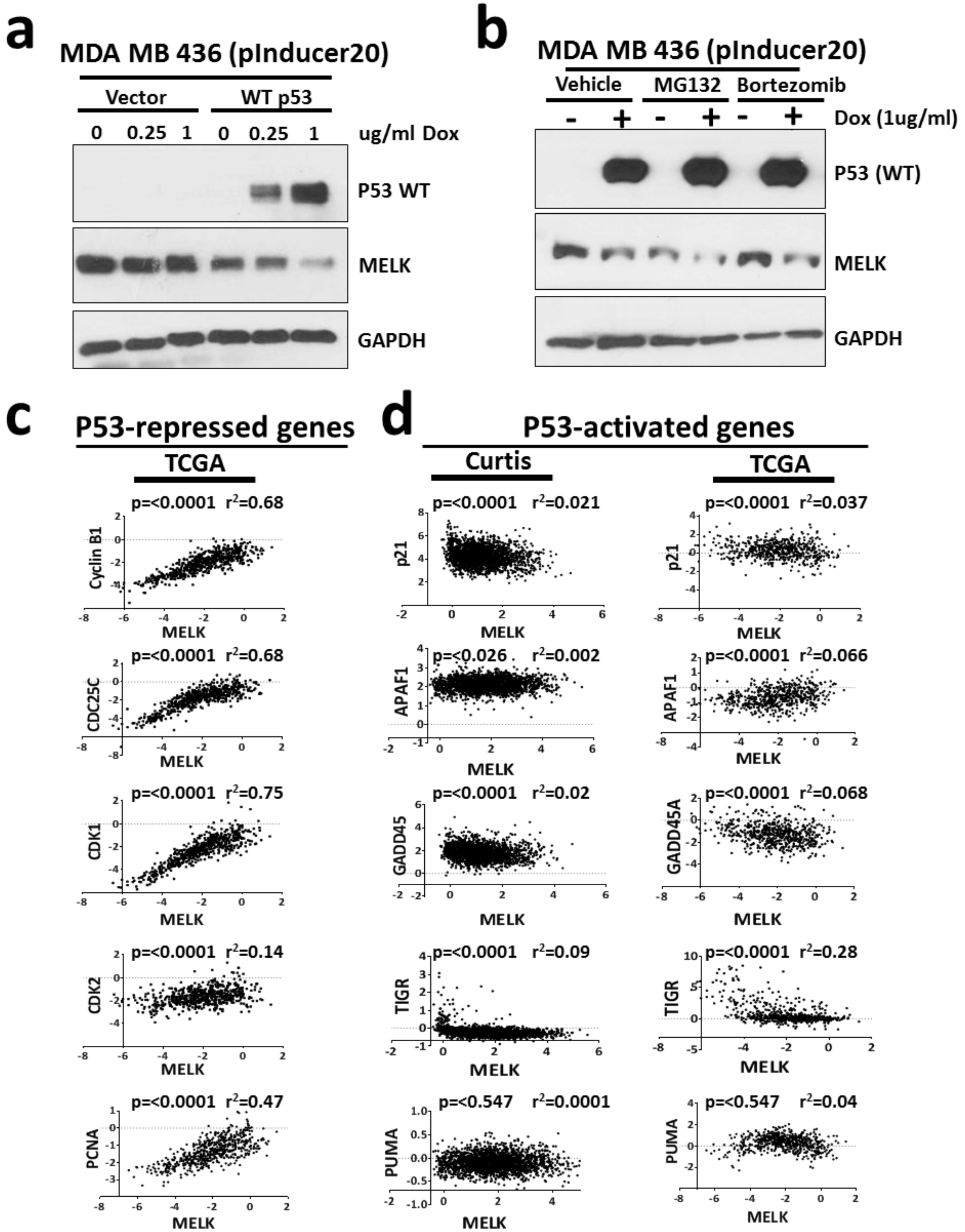


**Supplementary Figure 3**

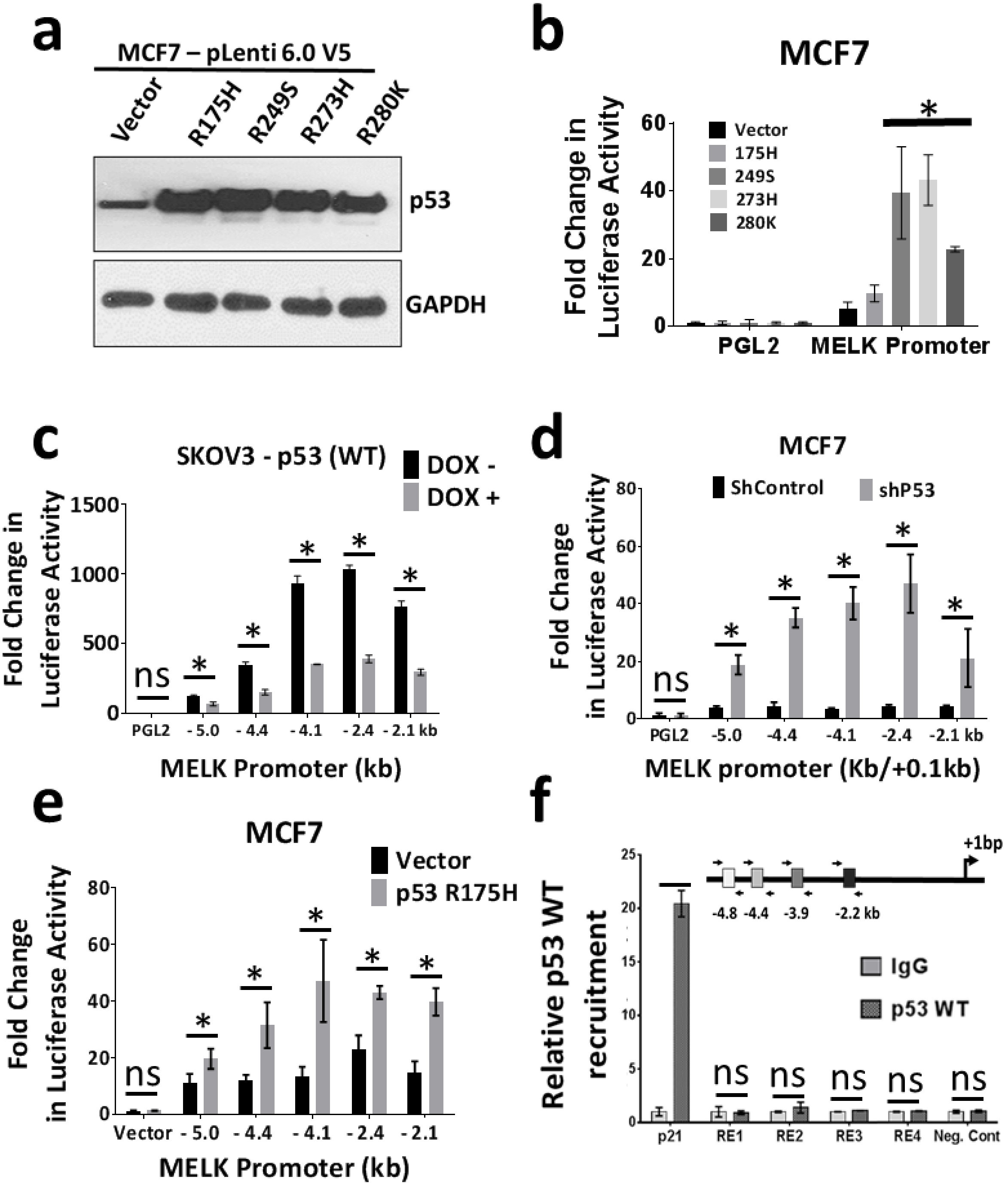


**Supplementary Figure 4**

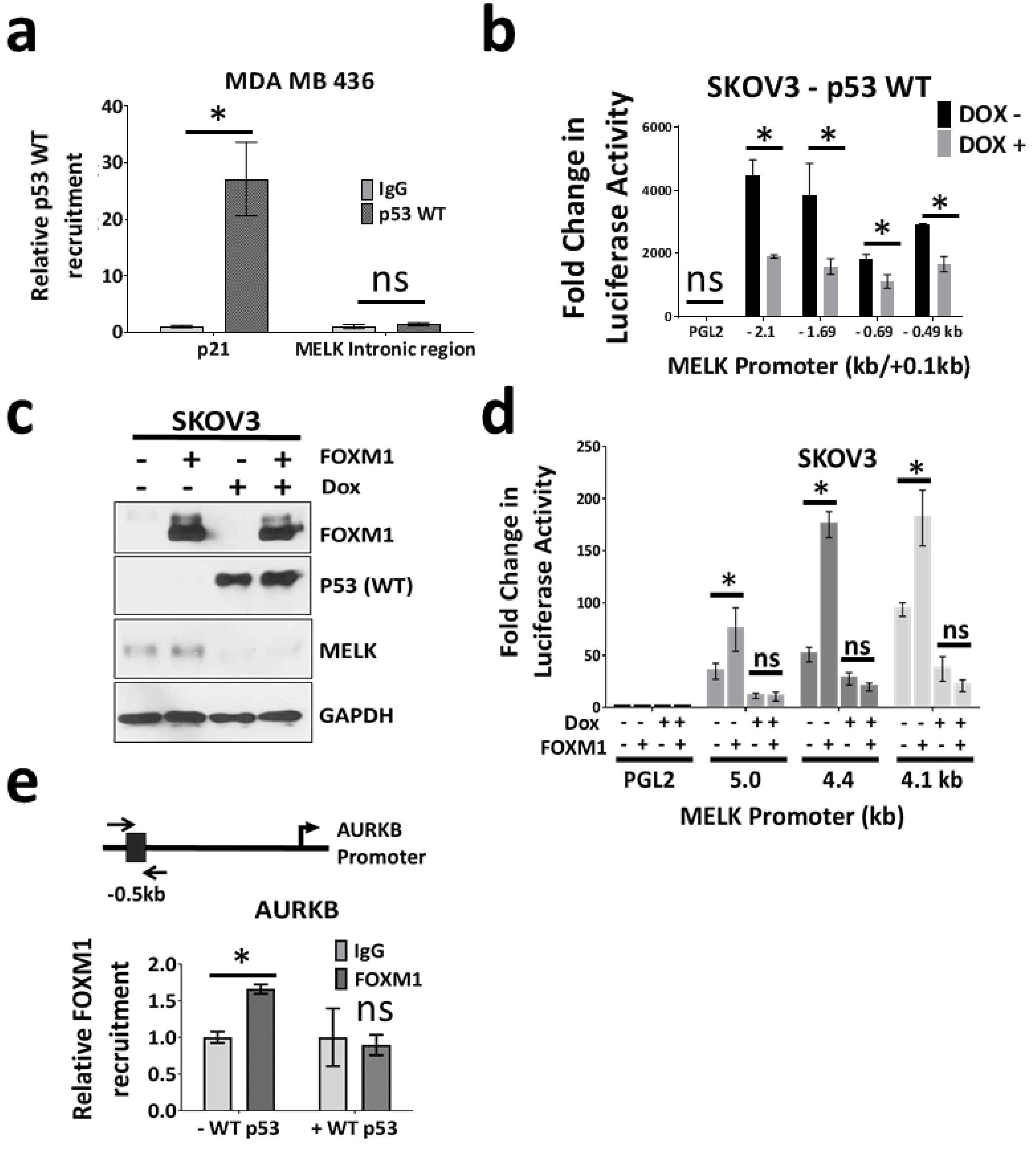


# Supplementary Figure 5

**Supplementary Figure 6.** (Raw data for the Western blots included in each figure and supplementary figure.)

# Figure 1

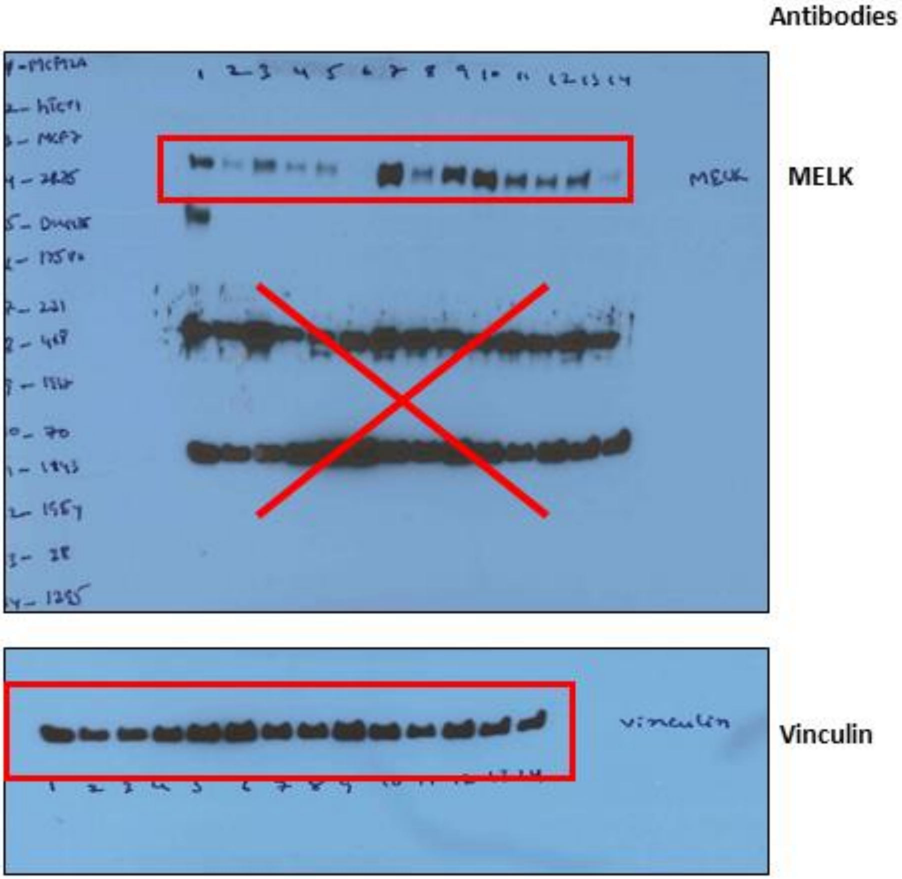

## Figure 2a

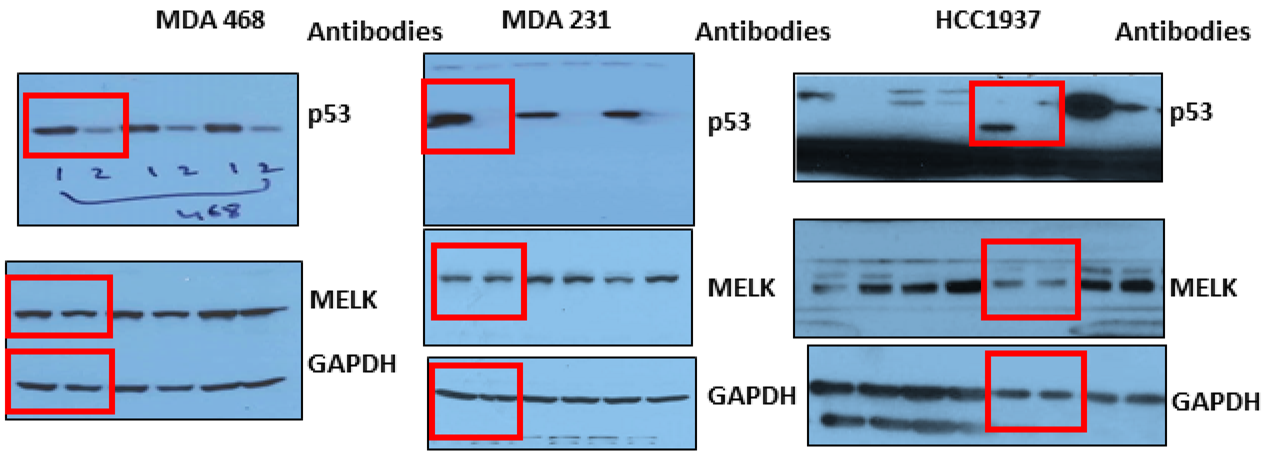


## Figure 2b

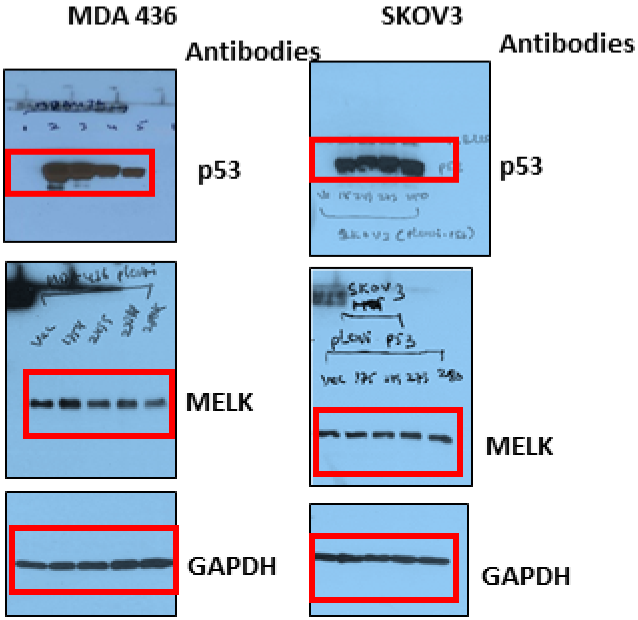


## Figure 2c

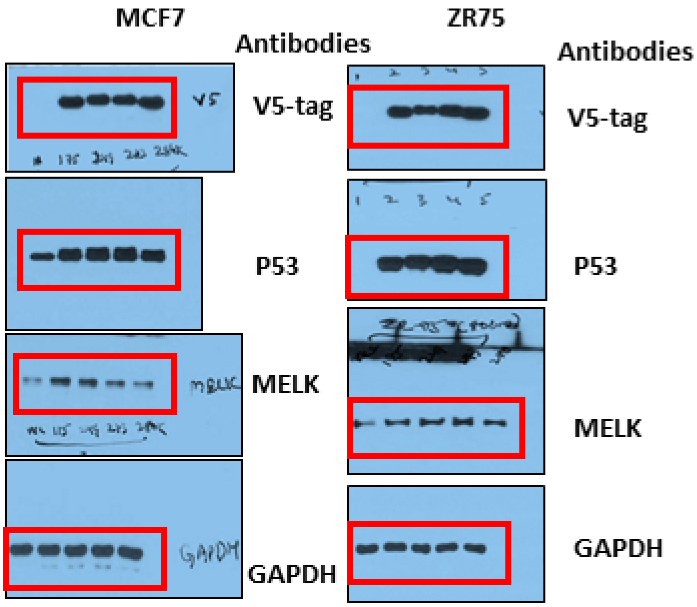

# Figure 2d

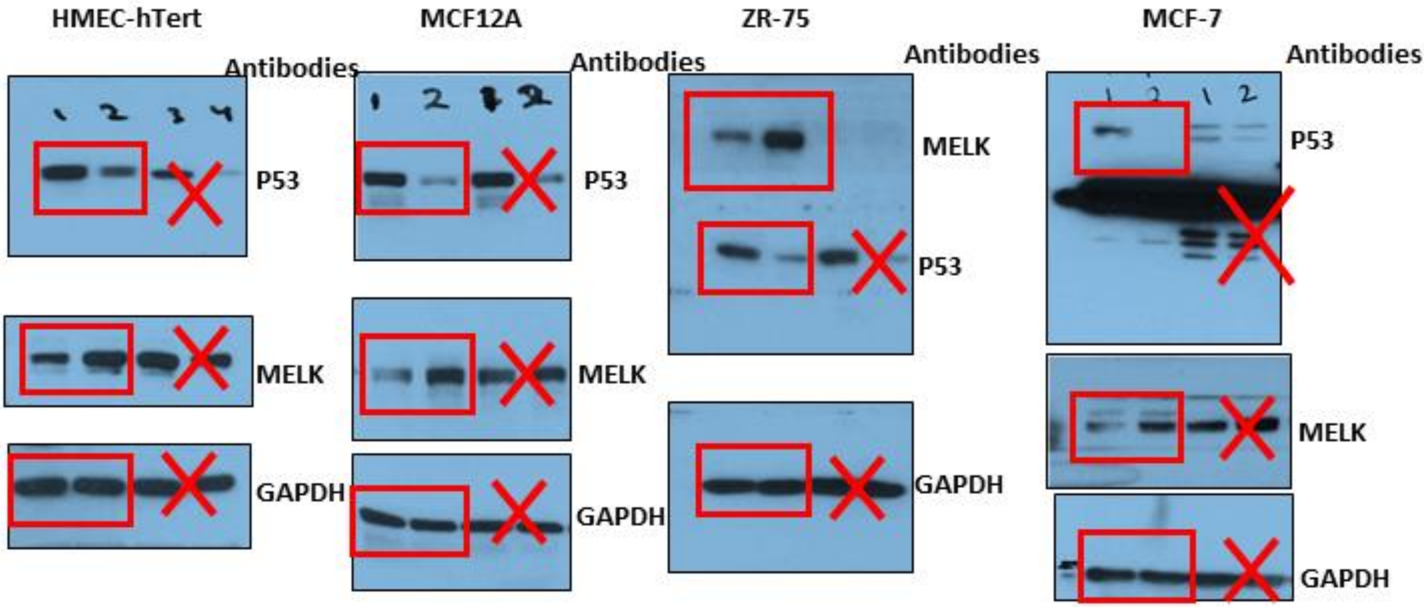

HMEC-hTert
MCF12A
ZR-75
MCF-7
Antibodies
Antibodies
Antibodies
Antibodies
P53
MELK
GAPDH
P53
MELK
GAPDH
MELK
P53
GAPDH
P53
MELK
GAPDH

## Figure 3a

MDA MB 436

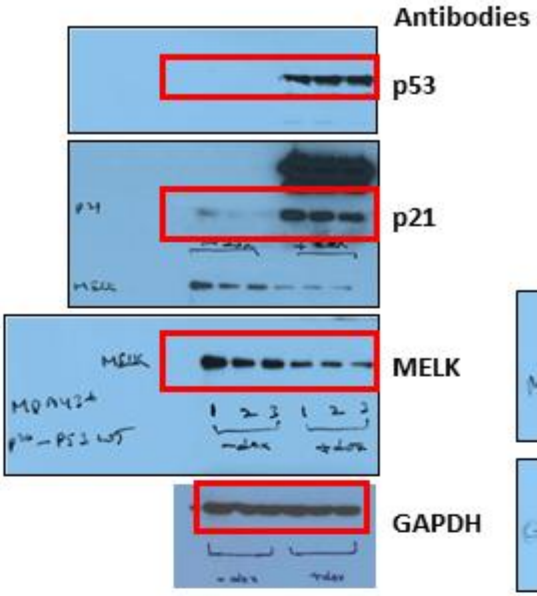


## Figure 3c

SKOV3

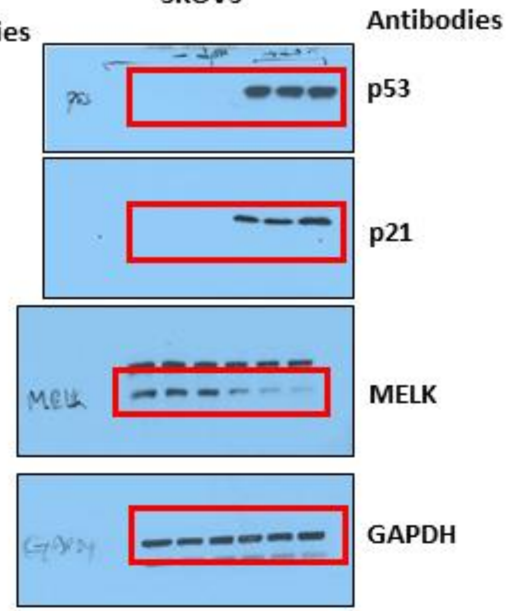


## Figure 3e

MCF7

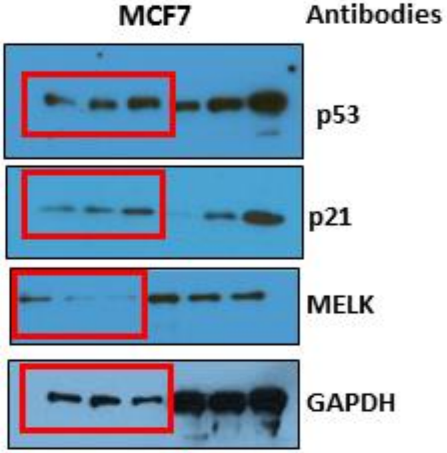


Cal51

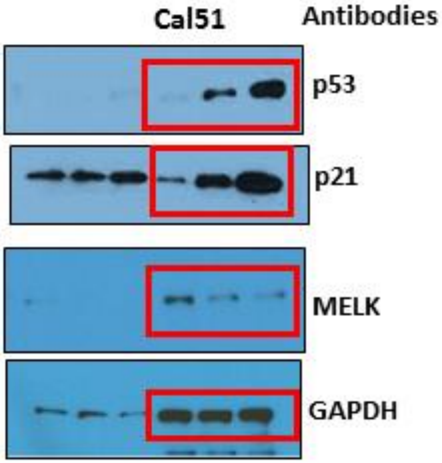

Figure 4b

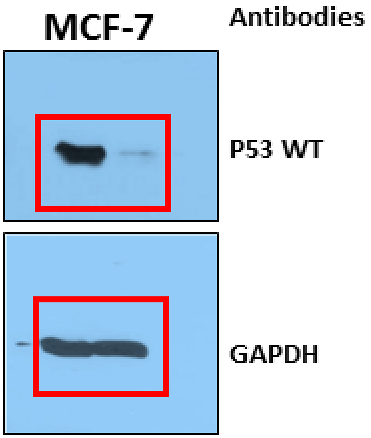


Figure 4c

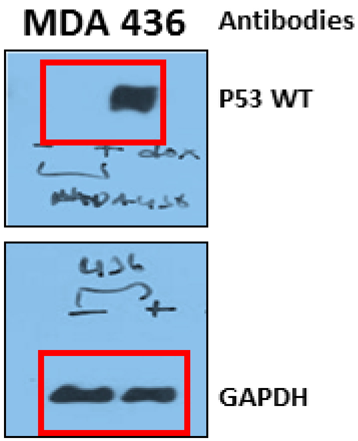


Figure 4d

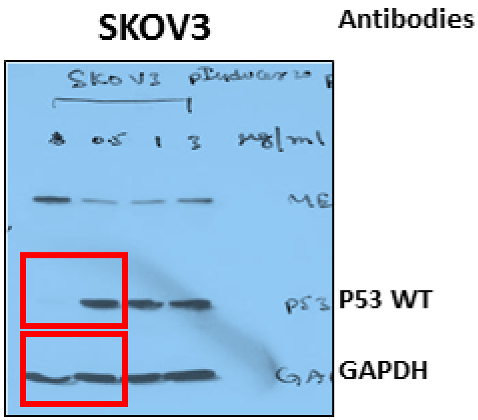

Figure 5a

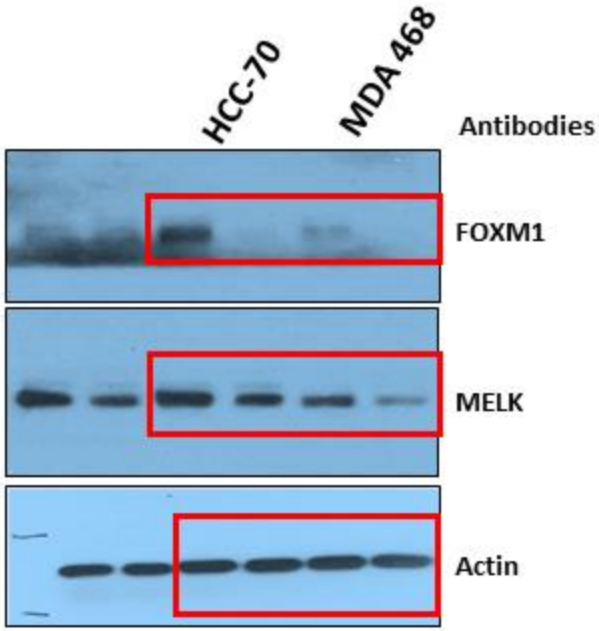


Figure 5b

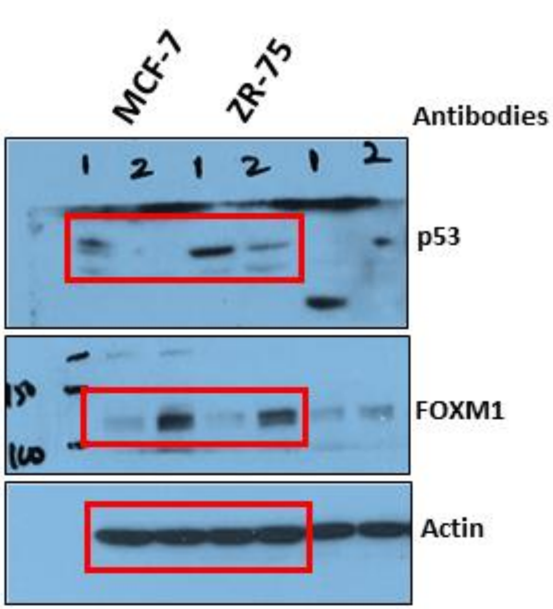


Figure 5c

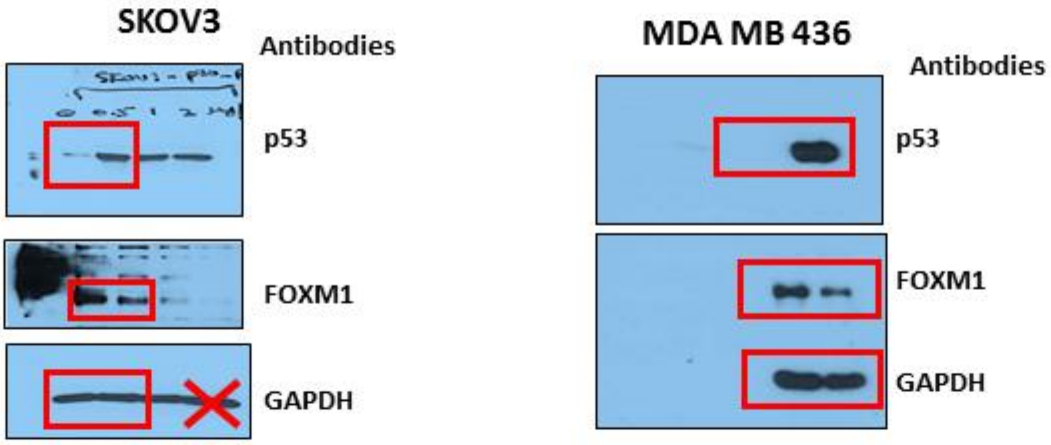

Figure 5e

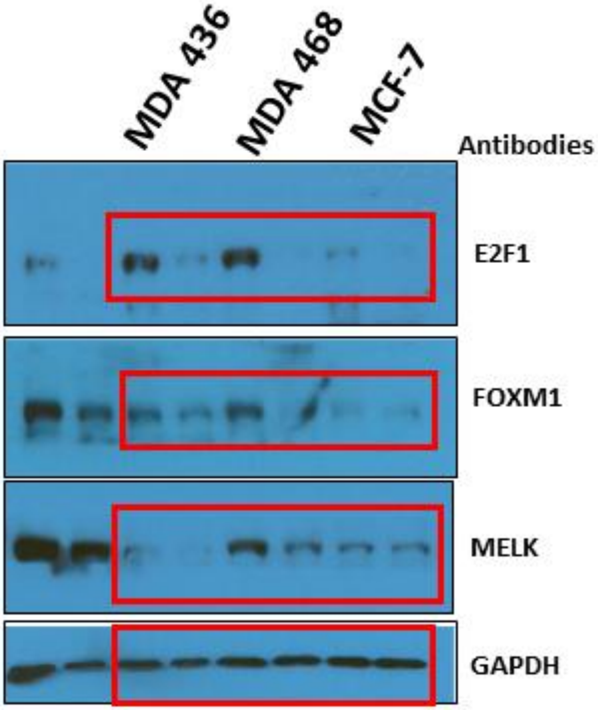


Figure 5f

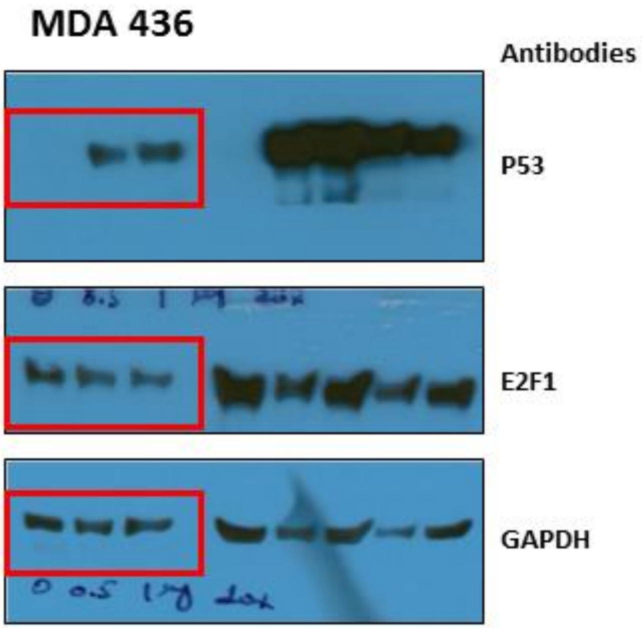

Figure 6a

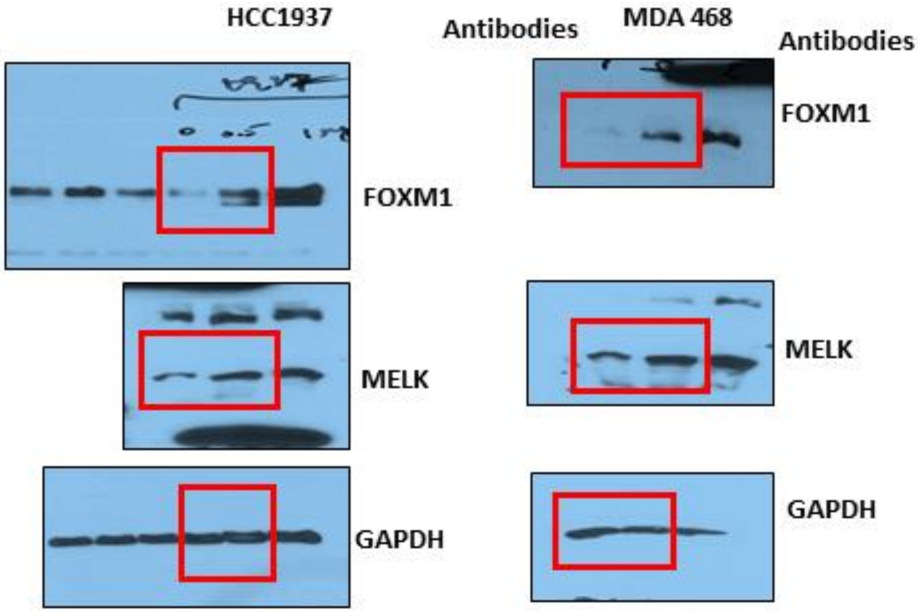


Figure 6b

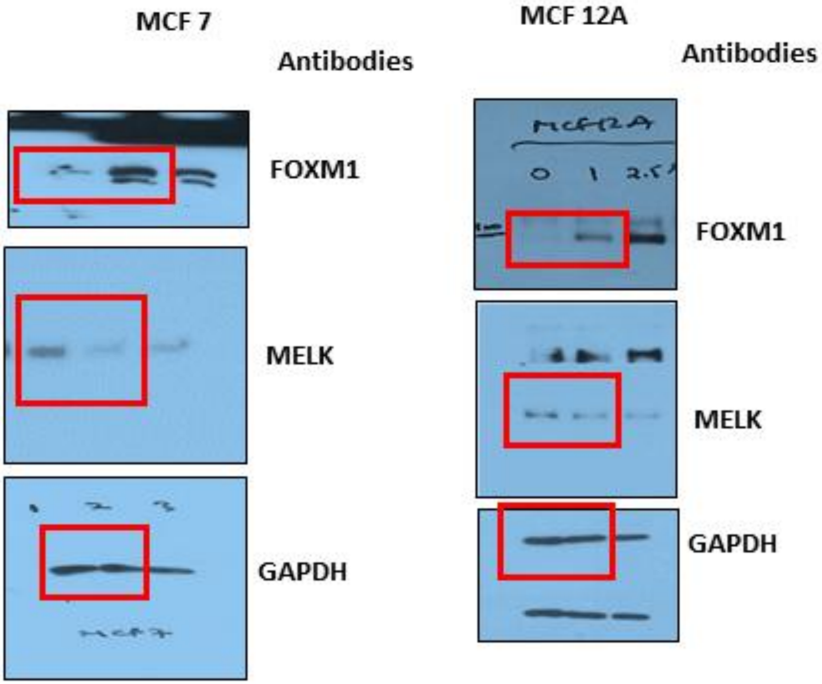

Figure 6c

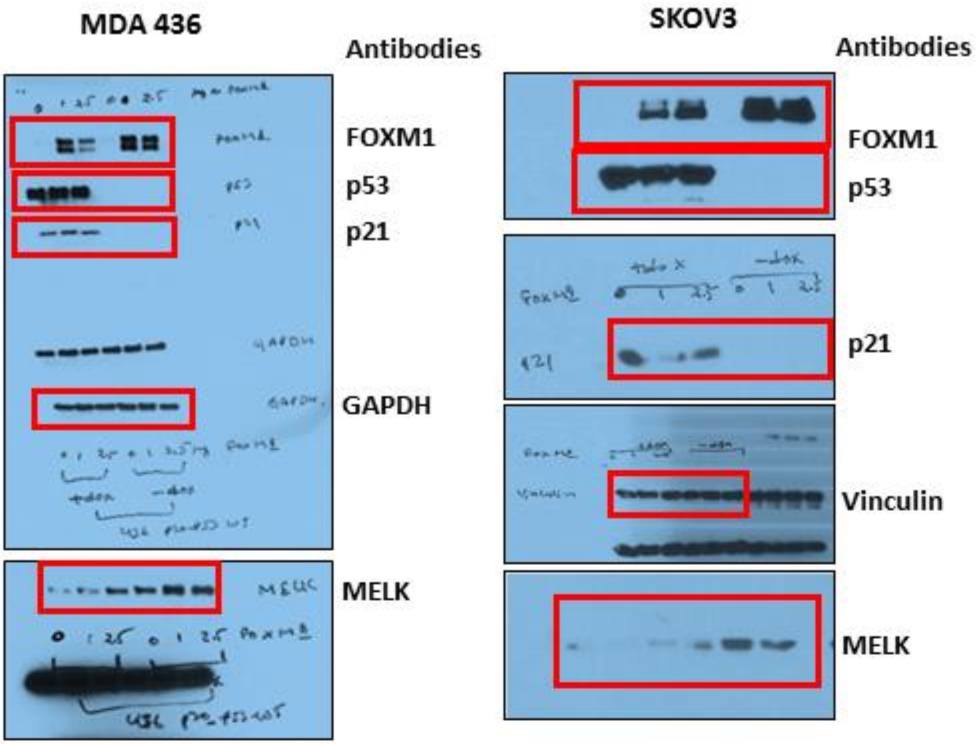


Figure 6d

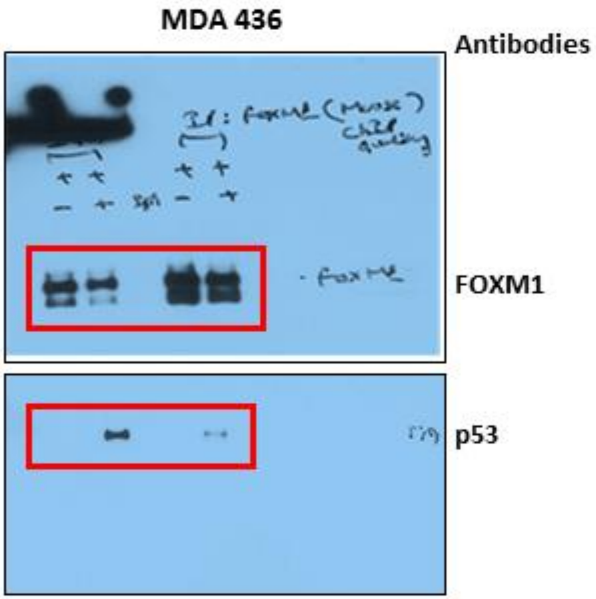

## Supplementary Figure 3a

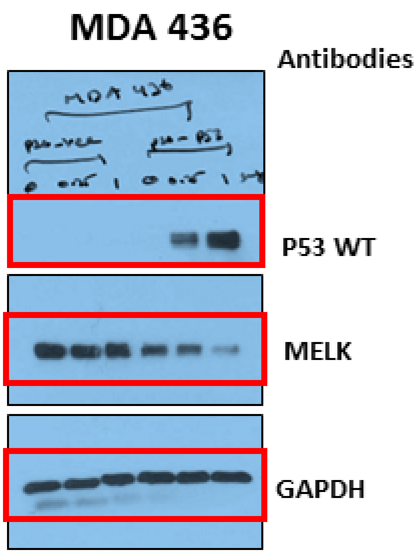


## Supplementary Figure 3b

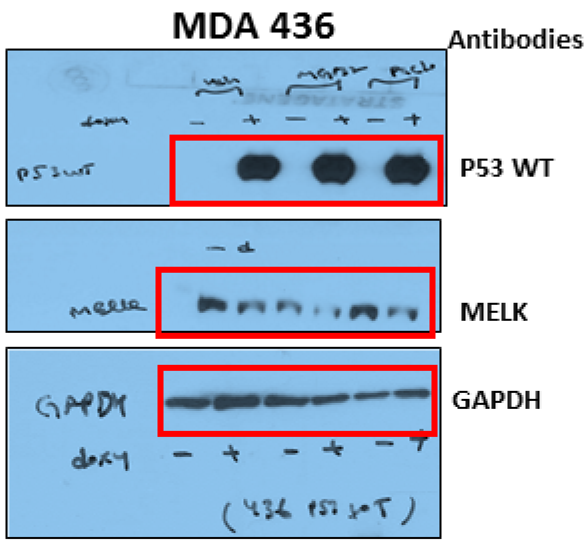


## Supplementary Figure 4a

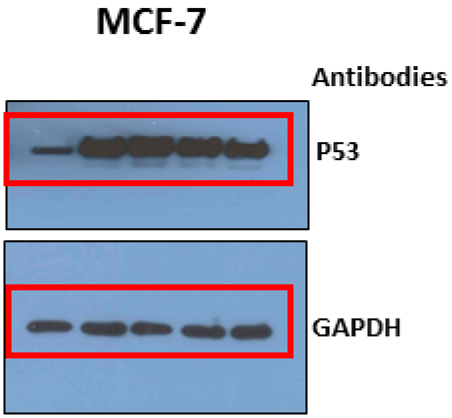


## Supplementary Figure 5c

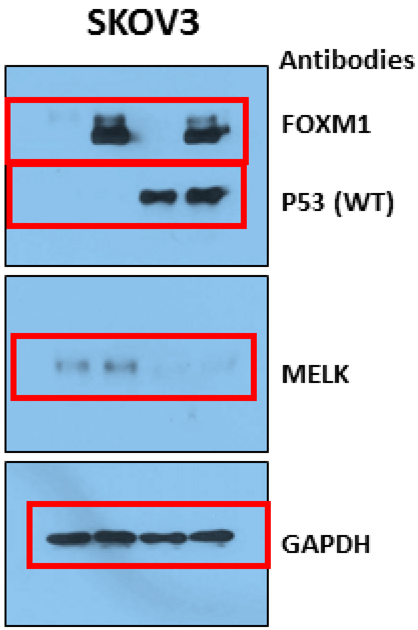

Supplement: Supplementary file 1 — Supplementary Information - Tables, Legends and Figures [file 41523_2019_143_MOESM1_ESM.pdf]
